# An Incomplete Spin Transition Associated with a *Z*′=1→*Z*′=24 Crystallographic Symmetry Breaking

**DOI:** 10.1002/chem.201704896

**Published:** 2017-11-22

**Authors:** Izar Capel Berdiell, Rafal Kulmaczewski, Oscar Cespedes, Malcolm A. Halcrow

**Affiliations:** ^1^ School of Chemistry University of Leeds Woodhouse Lane Leeds LS2 9JT United Kingdom; ^2^ School of Physics and Astronomy University of Leeds E.C. Stoner Building Leeds LS2 9JT United Kingdom

**Keywords:** iron, N ligands, spin-crossover, symmetry-breaking, X-ray crystallography

## Abstract

Crystalline [Fe*L*_2_][BF_4_]_2_ **⋅**Me_2_CO (*L=N*‐[2,6‐di{pyrazol‐1‐yl}pyrid‐4‐yl]acetamide) is high‐spin at room temperature, and undergoes an abrupt, hysteretic spin‐crossover at *T* ${}_{1/2}$ =137 K (Δ*T* ${}_{1/2}$ =14 K) that proceeds to about 50 % completeness. This is associated with a crystallographic phase transition, from phase 1 (*P*2_1_/*c*, *Z=*4) to phase 2 (*P*2_1_, *Z=*48). The cations associate into chains in the crystal through weak intermolecular π⋅⋅⋅π interactions. Phase 2 contains a mixture of high‐spin and low‐spin molecules, which are grouped into triads along these chains. The perchlorate salt [Fe*L*_2_][ClO_4_]_2_ **⋅**Me_2_CO also adopts phase 1 at room temperature but undergoes a different phase transition near 135 K to phase 3 (*P*2_1_/*c*, *Z=*8) without a change in spin state.

The structural chemistry of spin‐crossover (SCO) compounds^1^, ^2^, ^3^ continues to be heavily studied. The structural relationships underlying SCO functionality^4^ are fundamental to the de novo design of new SCO materials for device applications or in nanoscience.^3^, ^5^ Moreover, SCO crystals have proven especially useful for studying the fundamental physics of crystallographic phase transitions.^6^, ^7^

Crystallographic symmetry breaking during SCO is observed in a number of materials.^8^ Re‐entrant symmetry breaking can lead to an intermediate crystal phase during the SCO process, containing a mixture of high‐spin and low‐spin molecules in its asymmetric unit.^9^, ^10^, ^11^, ^12^, ^13^, ^14^ The resultant mixed spin‐state population is generally retained over a temperature range, before undergoing another phase change accompanied by full conversion to the low‐spin form. Alternatively, irreversible symmetry breaking can occur during SCO to a low‐temperature phase which can be either fully low‐spin,^15^ or contain a mixture of high‐ and low‐spin molecules as before.^16^, ^17^ Symmetry‐breaking involving a doubling of the crystallographic asymmetric unit is most common in either scenario, with the lower symmetry phase containing distinct high‐spin and low‐spin molecules arranged in a 0D, 1D, or 2D sublattice.^9^, ^15^, ^16^ However, SCO‐induced phase changes involving tripling,^10^, ^11^, ^17^ quadrupling,^12^ six‐fold,^13^ or 7.5‐fold expansion^14^ of the asymmetric unit have also been reported, leading to more complicated patterning of spin‐states in these low‐symmetry phases.

As part of our continuing investigations of complexes derived from [Fe(bpp)_2_]^2+^ (bpp=2,6‐di{pyrazol‐1‐yl}pyridine),^18^, ^19^ we now describe a material exhibiting cooperative but incomplete SCO, whose low‐temperature phase shows a 24‐fold expansion of the crystallographic asymmetric unit. As well as being the most dramatic example of SCO‐induced symmetry breaking yet reported, the low‐symmetry phase contains one of the largest numbers of crystallographically independent molecules (*Z*′) observed in a molecular compound.^20^

The new ligand *N*‐(2,6‐di{pyrazol‐1‐yl}pyrid‐4‐yl)acetamide (*L*) was prepared by treatment of 4‐amino‐2,6‐di{pyrazol‐1‐yl}pyridine^21^ with acetyl chloride. The reaction is sluggish, reflecting the de‐activated nature of the (pyrid‐4‐yl)amino group, but proceeds in 67 % yield if a 6.5x excess of acetyl chloride is used. The identity of *L* was confirmed crystallographically, which showed a complicated pattern of acetamido group disorder and intermolecular hydrogen bonding, associated with the partial occupancy of a lattice water molecule.^22^ Complexation of *L* with 0.5 equiv. Fe[BF_4_]_2_ **⋅**6 H_2_O or Fe[ClO_4_]_2_ **⋅**6 H_2_O in acetone afforded crystalline [Fe*L*_2_][BF_4_]_2_ **⋅**Me_2_CO (**1[BF_4_]_2_⋅**Me_2_CO) and [Fe*L*_2_][ClO_4_]_2_ **⋅**Me_2_CO (**1[ClO_4_]_2_⋅**Me_2_CO) after slow diffusion of diethyl ether vapor into the filtered reaction mixtures (Scheme 1). Samples of **1[BF_4_]_2_⋅**Me_2_CO and **1[ClO_4_]_2_⋅**Me_2_CO retain their solvent under ambient conditions by microanalysis, and are phase‐pure by X‐ray powder diffraction.^22^

**Scheme 1:**
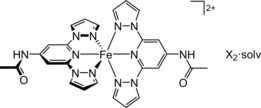
Compound **1X_2_⋅**solv (X^−^=BF_4_^−^ or ClO_4_^−^; solv=Me_2_CO).

Solid **1[BF_4_]_2_⋅**Me_2_CO is high‐spin at room temperature, but undergoes an incomplete spin transition on cooling according to magnetic susceptibility data (Figure 1). The transition is abrupt and shows a small thermal hysteresis loop, with *T* ${}_{1/2}$ ↓=130 and *T* ${}_{1/2}$ ↑=142 K (scan rate 5 K min^−1^) immediately below the transition temperature, *χ*_M_ *T*=2.0 cm^3^ mol^−1^ K, which corresponds to about a 41 % low‐spin population at that temperature. This slowly decreases to 1.7 cm^3^ mol^−1^ K (50 % low‐spin) upon further cooling to 95 K. Below 95 K the sample remains in a 1:1 high:low‐spin form, with an additional decrease in *χ*_M_ *T* below 50 K reflecting zero‐field splitting of the residual high‐spin content of the sample.^23^

**Figure 1:**
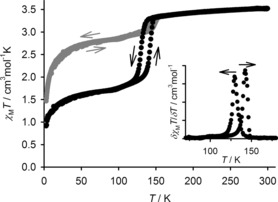
Variable temperature magnetic susceptibility data for polycrystalline **1[BF_4_]_2_⋅**Me_2_CO (black) and **1[ClO_4_]_2_⋅**Me_2_CO (gray), on a temperature ramp of 5 K min^−1^. The inset shows the first derivative of the data for **1[BF_4_]_2_⋅**Me_2_CO.

Crystals of **1[BF_4_]_2_⋅**Me_2_CO at 240 K adopt the monoclinic space group *P*2_1_/*c*, with one formula unit in the asymmetric unit (i.e. *Z=*4). The complex's metric parameters imply it is high‐spin at that temperature, as expected from the magnetic data. The compound associates into discrete {[FeL_2_][BF_4_]_2_} assemblies, through N−H⋅⋅⋅F hydrogen bonds between the acetamido substituents and BF_4_^−^ ions (which are all disordered at that temperature; Figure 2). The only significant contact between cations in the lattice is a weak intermolecular π⋅⋅⋅π overlap between pyrazolyl rings, of nearest neighbors related by translation along the crystallographic *a* direction.

**Figure 2:**
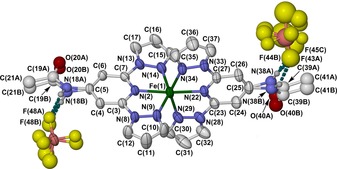
View of the {[Fe*L*_2_][BF_4_]_2_} hydrogen‐bonded assembly in phase 1 of **1[BF_4_]_2_⋅**Me_2_CO, showing the atom numbering scheme employed. All orientations of the disordered acetamido substituents and BF_4_^−^ ions are shown. Displacement ellipsoids are at the 50 % probability level, and C‐bound H atoms are omitted for clarity. Color code: C, white; H, pale gray; B, pink; F, yellow; Fe, green; N, blue; O, red.

Cooling the crystal below the SCO transition temperature caused the appearance of new, closely spaced diffraction spots,^22^ implying a transition to a lower symmetry phase (phase 2) with a large unit cell. Allowing for the change in spin states, the unit cell transformation to form phase 2 is *a*′=2 *c*, *b*′=*b*, *c*′=6 *a* and *β*′=*β*, giving *V*≈42 800 Å^3^ which is 12× larger than for phase 1. Variable temperature unit cell data show the phase 1↔phase 2 transition occurs at 135±5 K in cooling mode and 145±5 K in warming mode, which reproduces the thermal hysteresis in the magnetochemical transition.^22^

After several attempts from different crystals and diffractometers, a satisfactory refinement of phase 2 was achieved at 130 K, in the space group *P*2_1_ (*Z=*48). The loss of the crystallographic *c* glide and inversion center in phase 2, together with its unit cell volume expansion, generates 24 unique molecules in its asymmetric unit which are labelled ‘A′–‘X′ (Figure 3). The refinement of phase 2 is of moderate precision, reflecting the size of the model and the lower data resolution from the very large unit cell. However the main features of the structure are clear.

**Figure 3:**
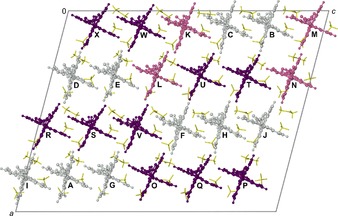
The asymmetric unit of the low‐temperature phase of **1[BF_4_]_2_⋅**Me_2_CO, superimposed on the crystallographic unit cell viewed parallel to the [0 1 0] crystal vector. High‐spin cations are colored white, low‐spin cations are purple and cations with a mixed high/low‐spin population are pink; anions and solvent (yellow) are de‐emphasized for clarity. The letter labels for each unique molecule in the model are also shown.^24^

Molecules A–J in the refinement are fully or predominantly high‐spin according to their metric parameters; molecules O–X are fully or predominantly low‐spin; and molecules K–N have a mixed high:low‐spin population at the temperature of measurement (Figure 3). That is consistent with the approximate 1:1 high:low‐spin ratio expected from the magnetic data (Figure 1). The same pattern of N−H⋅⋅⋅F hydrogen bonding occurs in phase 2 as in phase 1 although the acetamido substituents, and around half of the anions and solvent molecules, have become crystallographically ordered at the lower temperature.

As before, cations in the lattice associate by weak intermolecular π⋅⋅⋅π interactions into chains, which run parallel to the unit cell *c* axis in phase 2. The asymmetric unit contains four unique chains, whose molecules have a HS‐HS‐HS‐LS‐LS‐LS or HS‐HS‐MS‐LS‐LS‐MS (HS=high‐spin; LS=low‐spin; MS=mixed spin state population) spin‐state patterning. The four mixed‐spin molecules are well‐separated from each other in the lattice (Figure 3), and some or all of these might gradually increase their low‐spin population upon further cooling. That could explain the small additional decrease in *χ*_M_ *T* observed between 125 and 95 K (Figure 1).

The presence or absence of SCO in solid, high‐spin [Fe(bpp)_2_]^2+^ derivatives often correlates with their coordination geometry. This is conveniently expressed by the parameters *θ* (the dihedral angle between the least squares planes of the ligands) and *φ* (the *trans*‐N{pyridyl}‐Fe‐N{pyridyl} bond angle, which is N(2)‐Fe(1)‐N(22) in Figure 1).^18^, ^22^ An ideal *D*_2*d*_ symmetric complex gives *θ*=90° and *φ*=180°. Most low‐spin [Fe(bpp)_2_]^2+^ derivatives approach these values, but high‐spin complexes show much more variation. In practice, high‐spin complexes deviating more strongly from the ideal values of *θ* and *φ* are less likely to transform to their low‐spin state upon cooling.^18^, ^25^

Notably, nine of the ten high‐spin cations in phase 2 have a more distorted coordination geometry than the high‐spin molecule in phase 1, which could explain why they remain high‐spin at low temperatures (Figure 4). Interestingly, these follow a near‐linear *θ* versus *φ* relationship, which is not usual in plots of this type.^19^ That implies the high‐spin molecules all distort along the same structural pathway, which should be a function of the anisotropic plasticity of the crystal lattice. That is reasonable, since the molecules are all approximately co‐aligned in the lattice (Figure 3). All the low‐spin molecules, and three of the four mixed‐spin iron sites, have less distorted geometries than the phase 1 molecule as expected.

**Figure 4:**
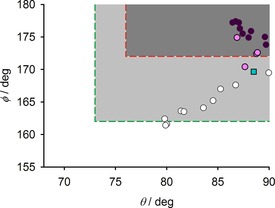
Molecular geometries of the cations in **1[BF_4_]_2_⋅**Me_2_CO. The cyan square is the phase 1 cation, while the phase 2 molecules are circles color coded as in Figure 3. High‐spin complexes in the shaded parts of the graph commonly (dark gray) or rarely (pale gray) exhibit SCO on cooling. High‐spin [Fe(bpp)_2_]^2+^ derivatives in the unshaded part of the graph never exhibit SCO in the solid state.^18^, ^25^

Crystalline **1[ClO_4_]_2_⋅**Me_2_CO also adopts high‐spin phase 1 at room temperature, and a full structure refinement at 170 K showed only minor differences to this phase with the BF_4_^−^ salt. However, no SCO was observed upon cooling **1[ClO_4_]_2_⋅**Me_2_CO to 100 K on the diffractometer. Rather, at 135±5 K the crystals transform to a new phase (phase 3), which retains the *P*2_1_/*c* space group but with a doubled unit cell *a* dimension (as well as small increases in *c* and *β*).^22^ Both unique molecules in phase 3, labelled ‘A′ and ‘B′, are fully high‐spin from their metric parameters, with molecule B showing significantly reduced *θ* and *φ* values compared to phase 1.^22^ The π⋅⋅⋅π‐stacked cation chains, which now run parallel to the unit cell *a* axis, contain alternating A and B cations.

Magnetic susceptibility data confirmed that **1[ClO_4_]_2_⋅**Me_2_CO indeed remains predominantly high‐spin between 5–300 K. However, an abrupt reduction of *χ*_M_ *T* from 3.3 to 3.0 cm^3^ mol^−1^ K occurs reproducibly near 145 K, close to the crystallographic phase transition temperature (Figure 1). For a phase change to have such an effect on *χ*_M_ *T*, without an associated spin transition, is unusual in a compound of this type.^26^ However high‐spin [Fe(bpp)_2_]^2+^ derivatives with reduced values of *θ* and *φ*, as in molecule B of phase 3,^22^ can exhibit magnetic moments up to 10 % lower than their undistorted analogues.^27^ Hence, rather than indicating a change in spin‐state population, the magnetochemical feature at 145 K might simply reflect the changes in molecular coordination geometry during the high‐spin phase 1→phase 3 transition.

In conclusion, thermal SCO in **1[BF_4_]_2_⋅**Me_2_CO yields a low temperature phase with an approximate 1:1 high:low‐spin population, that is distributed between 24 crystallographically unique molecules (i.e. *Z*′=24^20^). This is the most severe example of symmetry breaking yet observed in an SCO crystal.^8^ Moreover, notwithstanding one compound with *Z*′=56,^28^ crystals with such high *Z*′ values as phase 2 are very rare.^20^, ^29^ High *Z*′ crystals have been proposed to be kinetic intermediates in the crystallization pathway; or, to arise from frustrated, mutually orthogonal packing interactions in the lattice.^20^ Either description could apply to phase 2. On one hand, phase 2 may be an intermediate in the SCO of **1[BF_4_]_2_⋅**Me_2_CO, with around half the molecules kinetically trapped in their high‐spin form.^10^, ^30^ On the other, competing ferroelastic and antiferroelastic interactions between molecules over different length scales in the lattice, are also known to stabilize mixed‐spin phases in SCO materials.^31^

## Experimental Section

Synthetic procedures, crystallographic data, and details of the instrumentation used for the spectroscopic and crystal structure measurements are given in the Supporting Information.^22^

## Conflict of interest

The authors declare no conflict of interest.

## Supporting information

As a service to our authors and readers, this journal provides supporting information supplied by the authors. Such materials are peer reviewed and may be re‐organized for online delivery, but are not copy‐edited or typeset. Technical support issues arising from supporting information (other than missing files) should be addressed to the authors.

Supplementary
