# Supplementary material for: An Incomplete Spin Transition Associated with a Z′=1→Z′=24 Crystallographic Symmetry Breaking
Source: Chemistry. 2017 Nov 22;24(20):5055–9. doi: 10.1002/chem.201704896 (PMC5972820; doi:10.1002/chem.201704896)
Supplement: Supplementary file 1 — Supplementary [file CHEM-24-5055-s001.pdf]

CHEMISTRY
A European Journal

Supporting Information

An Incomplete Spin Transition Associated with a $Z' = 1 \rightarrow Z' = 24$ Crystallographic Symmetry Breaking

Izar Capel Berdiell,[a] Rafal Kulmaczewski,[a] Oscar Cespedes,[b] and Malcolm A. Halcrow*[a]

chem_201704896_sm_miscellaneous_information.pdf

		Page
**Experimental Details**		2
**Table S1**	Experimental data for the crystal structures in this work.	4
**Figure S1**	^{1}H and ^{13}C NMR spectra of *L*.	5
**Figure S2**	Crystallographic asymmetric unit of bpp^{NH2}.	6
**Figure S3**	The puckered 2D hydrogen bond network in bpp^{NH2}.	6
**Figure S4**	Crystallographic asymmetric unit of $L\cdot¼H_2O$.	7
**Figure S5**	A hydrogen-bonded $[L_4(H_2O)]$ tetrad in $L\cdot{}^1/_4H_2O$.	7
**Table S2**	Hydrogen bond parameters for the organic ligand structures in this work.	8
**Definitions of the Structural Parameters in Tables S3 and S6**		9
**Scheme S1**	Angles used in the definitions of the coordination distortion parameters Σ and Θ.	9
**Scheme S2**	Definition of the Jahn-Teller distortion parameters θ and ϕ.	9
**Table S3**	Bond distances and metric parameters for **1[BF$_4$]$_2$**$\cdot Me_2CO$ at different temperatures.	10
**Figure S6**	Diffraction images from phase 1 and phase 2 of **1[BF$_4$]$_2$**$\cdot Me_2CO$ in the *hk*0 and *h*0*l* zones.	12
**Figure S7**	The $[FeL_2][BF_4]_2$ assembly in phase 1 of **1[BF$_4$]$_2$**$\cdot Me_2CO$.	13
**Figure S8**	The twenty-four unique $[FeL_2][BF_4]_2$ moieties in phase 2 of **1[BF$_4$]$_2$**$\cdot Me_2CO$.	14
**Table S4**	Hydrogen bond parameters for phases 1 and 2 of **1[BF$_4$]$_2$**$\cdot Me_2CO$.	17
**Table S5**	Intermolecular $\pi\ldots\pi$ contacts in phases 1 and 2 of **1[BF$_4$]$_2$**$\cdot Me_2CO$.	18
**Figure S9**	The asymmetric unit and the full unit cell contents of phase 2 of **1[BF$_4$]$_2$**$\cdot Me_2CO$.	19
**Table S6**	Bond distances and metric parameters for **1[ClO$_4$]$_2$**$\cdot Me_2CO$ at different temperatures.	20
**Figure S10**	The unique $[FeL_2][ClO_4]_2$ moieties in phases 1 and 3 of **1[ClO$_4$]$_2$**$\cdot Me_2CO$.	20
**Figure S11**	Diffraction images from phase 1 and phase 3 of **1[ClO$_4$]$_2$**$\cdot Me_2CO$ in the *hk*0 and *h*0*l* zones.	21
**Figure S12**	Plotted molecular geometries of the cations in phases 1 and 3 of **1[ClO$_4$]$_2$**$\cdot Me_2CO$.	22
**Table S7**	Hydrogen bond parameters for phases 1 and 3 of **1[ClO$_4$]$_2$**$\cdot Me_2CO$.	22
**Table S8**	Intermolecular $\pi\ldots\pi$ contacts in phases 1 and 3 of **1[ClO$_4$]$_2$**$\cdot Me_2CO$.	22
**Figure S13**	One layer of molecules and a full packing diagram of phase 3 **1[ClO$_4$]$_2$**$\cdot Me_2CO$.	23
**Figure S14**	Variable temperature unit cell parameters for **1[BF$_4$]$_2$**$\cdot Me_2CO$.	24
**Figure S15**	Variable temperature unit cell parameters for **1[ClO$_4$]$_2$**$\cdot Me_2CO$.	25
**Table S9**	Variable temperature unit cell data for **1[BF$_4$]$_2$**$\cdot Me_2CO$ and **1[ClO$_4$]$_2$**$\cdot Me_2CO$.	26
**Figure S16**	Room temperature X-ray powder diffraction patterns for the complexes in this study.	27
**References**		28

Experimental

Instrumentation

Magnetic susceptibility measurements were performed with freshly isolated, unground polycrystalline samples, using a Quantum Design SQUID/VSM magnetometer in an applied field of 5000 G and a temperature ramp of 5 $Kmin^{-1}$. A drop of solvent was added to the sample holder capsules to minimize desolvation of the samples during the measurement. Diamagnetic corrections for the samples were estimated from Pascal's constants;[1] a previously measured diamagnetic correction for the sample holder was also applied to the data.

Elemental microanalyses were performed by the London Metropolitan University School of Human Sciences microanalytical service. ^{1}H and ^{13}C NMR spectra were obtained using a Bruker Avance 400 spectrometer, operating at 400.1 and 100.6 MHz respectively. Electrospray mass spectra (ESMS) were obtained on a Bruker MicroTOF spectrometer, from MeCN feed solutions. All mass peaks have the correct isotopic distributions for the proposed assignments. Room temperature X-ray powder diffraction measurements were obtained from a Bruker D2 Phaser diffractometer, using Cu-K_α radiation (λ = 1.5419 Å).

Materials and methods

4-Amino-2,6-di(pyrazol-1-yl)pyridine (bpp^{NH2})[2] was prepared as previously described.[3] Other reagents and solvents were purchased commercially and used as supplied. All reactions were carried out in air using as-supplied AR-grade solvents.

Synthesis of *N*-(2,6-di{pyrazol-1-yl}pyrid-4-yl)acetamide (*L*). Triethylamine (0.22 g, 2.2 mmol) was added to a suspension of bpp^{NH2} (0.20 g, 0.89 mmol) in dichloromethane (35 cm^3). A solution of acetyl chloride (0.21 g, 2.7 mmol) in dichloromethane (5 cm^3) was then added, and the mixture was stirred for 1 hr at room temperature. Additional triethylamine (0.37 g, 3.6 mmol) was then carefully added, upon which the solution turned red/orange and a vapor was vigorously released. After the reaction subsided the solvent was removed and water (25 cm^3) was added to the residue. The resultant pale brown precipitate was extracted with chloroform and dried with $MgSO_4$. The crude material was purified by column flash chromatography (1:1 hexane:ethyl acetate eluent, Rf 0.31) yielding a white solid. Yield 0.16 g, 67 %. m.p. 210-212 °C; ^{1}H NMR ([D]chloroform): δ = 2.25 (s, 3H, CH_3), 6.48 (s, 2H, Pz H^4), 7.74 (s, 2H, Pz H^3), 7.98 (br s, 1H, N*H*), 8.04 (s, 2H, Py $H^{3/5}$), 8.53 (d, 2.3 Hz, 2H, Pz H^5); ^{13}C NMR ([D]chloroform): δ = 24.8 (1C, CH_3) 99.1 (2C, Py $C^{3/5}$), 107.9 (2C, Pz C^4), 127.2 (2C, Pz C^5), 142.3 (2C, Pz C^3), 149.4 (1C, Py C^4), 151.1 (2C, Py $C^{2/6}$), 168.8 (1C, *C*=O); ESMS *m/z* 269.1 $[HL]^+$, 291.1 $[NaL]^+$, 559.2 $[NaL_2]^+$; elemental analysis calcd (%) for $C_{13}H_{12}N_6O$ (268.28) C 58.2, H 4.51, N 31.3. Found (%) C 58.1, H, 4.70 N, 30.9.

Colourless crystals of $L\cdot¼H_2O$ were obtained from undried $CDCl_3$, upon slow evaporation of an NMR sample.

Synthesis of $[FeL_2][BF_4]_2\cdot Me_2CO$ ($\mathbf{1}[BF_4]_2\cdot Me_2CO$). Separate solutions of *L* (25 mg, 0.093 mmol) and $Fe[BF_4]_2\cdot 6H_2O$ (16 mg, 0.047 mmol) in acetone (2x 5 cm^3) were mixed, causing the solution to become an intense yellow color. The resultant mixture was concentrated to 5 cm^3 volume, then crystallized by slow diffusion of diethyl ether vapor to yield a yellow crystalline solid. Yield 27 mg, 70 %. Elemental analysis calcd (%) for $C_{26}H_{24}B_2F_8FeN_{12}O_2\cdot(CH_3)_2CO$ (824.09) C 42.3, H 3.70, N 20.4. Found (%) C 42.2, H, 3.53 N, 20.2.

Synthesis of $[FeL_2][ClO_4]_2\cdot Me_2CO$ ($\mathbf{1}[ClO_4]_2\cdot Me_2CO$). Method as for $\mathbf{1}[BF_4]_2\cdot Me_2CO$, using $Fe[ClO_4]_2\cdot 6H_2O$ (0.017 g, 0.047 mmol). Yield 36 mg, 90 %. ^{1}H NMR ([D_3]acetonitrile): δ = 1.2 (s, 6H, CH_3), 2.1 (s, 6H, {CH_3}$_2$CO), 45.0 and 46.6 (both 4H, Pz H^3 and Py $H^{3/5}$), 64.3 and 74.5 (both s, 4H, Pz H^4 and H^5); ESMS *m/z* 296.1 $[FeL_2]^{2+}$, 691.2 $[FeL_2ClO_4]^+$; elemental analysis calcd (%) for $C_{26}H_{24}Cl_2FeN_{12}O_{10}\cdot(CH_3)_2CO$ (849.38) C 41.0, H 3.56, N 19.8; found (%) C 40.9, H 3.43, N, 19.9.

WARNING Although we encountered no issues in handling $\mathbf{1}[ClO_4]_2$ during this study, metal/organic perchlorates are potentially explosive and should be handled with care in small quantities.

Single crystal X-ray structure determinations

All diffraction data were collected with an Agilent Supernova dual-source diffractometer, using monochromated Cu-K_α radiation (λ = 1.54184 Å). The diffractometer is fitted with an Oxford Cryosystems low-temperature device. Experimental details of the structure determinations are given in Table S1. A structures were solved by direct methods (*SHELXS97*[4]), and developed by full least-squares refinement on F^2 (*SHELXL97*,[4] *via X-SEED*[5] or *Olex2*[6]). Crystallographic figures were prepared using *X-SEED*,[5] and octahedral coordination volumes (V_{Oh}) were calculated with *Olex*2.[6]

Unless otherwise stated in the following paragraphs, all fully occupied non-H atoms in these structures were refined anisotropically, and H atoms were placed in calculated positions and refined using a riding model. Disordered anions were modelled using refined bond length and angle restraints, while disordered solvent and acetamido [NHC(O)Me] groups were treated with fixed C–C, C–N and C=O distance restraints.

Structure refinement details for bpp^{NH2}. No disorder is present in this structure, and no restraints were applied to the refinement. All H atoms were located in the Fourier map and allowed to refine freely. CCDC 1569541

Structure refinement details for *L*·¼H_2O. The asymmetric unit contains two formula units of the compound, with two molecules of the heterocycle on general crystallographic sites, and a half-occupied water molecule near the inversion centre ½, $^3/_2$, ½. The water molecule O(41) is only 2.164(4) Å from its symmetry equivalent related by 1–*x*, 3–*y*, 1–*z*, which is too close for them both to be occupied simultaneously. The acetamido substituents of both organic molecules are disordered over two half-occupied orientations, which were modelled without restraints. This disorder can be correlated with the intermolecular hydrogen bonding in the presence or absence of the water half-molecule.

Ordered and disordered non-H atoms in the model were refined anisotropically. Crystallographically ordered H atoms were located in the Fourier map and allowed to refine, with U_{iso} = 1.2xU_{eq}{C}. Not all the half occupied C- and N-bound H atoms were directly observed, so these were placed in calculated positions and refined using a riding model. The half-water H atoms were present in the Fourier map and refined, with the restraint O–H = 0.90(2) Å and with U_{iso} = 1.5xU_{eq}{O}. CCDC 1569546

Structure refinement details for 1[BF_4]$_2$·Me_2CO. At 240 K, the asymmetric unit of phase 1 contains one formula unit of the compound, with each moiety on a general crystallographic site. Both BF_4^- ions are disordered, one over two equally occupied sites that share a common B atom; and the other over three sites of occupancy 0.40:0.40:0.20, of which the first two also share a B atom. The acetone molecule is also disordered over three orientations (occupancies 0.60, 0.25 and 0.15), while both acetamido groups are also disordered over two half-occupied sites.

At 130 K the crystal has transformed to phase 2, with an asymmetric unit containing 24 unique formula units of the compound: that is, 24 complex cations, 48 BF_4^- anions and 24 acetone molecules. No disorder was detected in the cations but 17 BF_4^- ions and 8 acetone molecules were modelled as disordered over two sites, with occupancy ratios appropriate to their displacement parameters. All fully occupied Fe, N, O and F atoms were refined anisotropically in the final least squares cycles; nine N atoms in the model required *ISOR* restraints to avoid becoming non-positive definite. The C and B atoms were left isotropic to preserve a reasonable observed data:parameter ratio in the refinement.

The high *Z*' value and space group ($P2_1$) of phase 2 imply that a transformation to a higher symmetry might be possible. However *ADSYMM*[7] analyses of the full model, and of a model based on the metal ion positions only, both favoured this choice of space group (the structure is too large for a *NEWSYMM* analysis[7]).

CCDC 1569542-1569543

[A structure analysis of phase 2 was also undertaken at station I19 of the Diamond synchrotron. While this reproduced the results reported here, the synchrotron dataset was of lower quality with only 45 % observed data to 2θ = 49.6° (λ = 0.6892 Å). So this refinement is not reported in detail, and has not been deposited with the CCDC. Experimental details for the synchrotron dataset: monoclinic, $P2_1$, *a* = 41.1091(4), *b* = 20.7084(2), *c* = 51.6438(6) Å, β = 104.952(1)°, *V* = 42476.0(8) Å^3, *Z* = 48, *T* = 100(2) K, D_{calc} = 1.545 gcm^{-3}, μ = 0.520 mm^{-1}, 553670 measured reflections, 163544 unique reflections, 72896 observed reflections ($I > 4\sigma(I)$), R_{int} = 0.079, R_1 = 0.164, wR_2 = 0.421, Flack parameter 0.501(14)].

Structure refinements of 1[ClO_4]$_2$· Me_2CO. The phase 1 structure at 170 K is isostructural to the high-temperature phase of the BF_4^- salt of this solvate. Both ClO_4^- ions in the model are disordered over two equally occupied sites, the acetone molecule is disordered over two orientations (occupancies 0.70 and 0.30), and acetamido substituent N(38)-C(41) is also disordered over two half-occupied sites. All fully occupied non-H atoms plus the half-occupied Cl disorder sites were refined anisotropically.

The asymmetric unit of phase 3 at 120 K contains two formula units of the compound, with two complex cations, four ClO_4^- anions and two acetone molecules lying on general crystallographic sites. One ClO_4^- ion is disordered, and was modelled over two sites with a 0.67:0.33 occupancy ratio. A 0.15-occupied minor disorder site was also resolved for one of the acetone molecules. All fully occupied non-H atoms plus the 0.67-occupied Cl atom were refined anisotropically.

CCDC 1569544-1569545.

Table S1 Experimental data for the crystal structures in this work.

	bpp^{NH2}	*L*·¼H_2O	**1[BF$_4$]$_2$**·Me_2CO		**1[ClO$_4$]$_2$**·Me_2CO	
			Phase 1	Phase 2	Phase 1	Phase 3
T [K]	120(2)	120(2)	240(2)	130(2)	170(2)	120(2)
Molecular formula	$C_{11}H_{10}N_6$	$C_{13}H_{12.50}N_6O_{1.25}$	$C_{29}H_{30}B_2F_8FeN_{12}O_3$	$C_{29}H_{30}B_2F_8FeN_{12}O_3$	$C_{29}H_{30}Cl_2FeN_{12}O_{11}$	$C_{29}H_{30}Cl_2FeN_{12}O_{11}$
M_r	226.25	272.79	824.12	824.12	849.40	849.40
Crystal system	orthorhombic	monoclinic	monoclinic	monoclinic	monoclinic	monoclinic
Space group	*Pbca*	*C*2/*c*	$P2_1/c$	$P2_1$	$P2_1/c$	$P2_1/c$
a [Å]	14.6350(3)	25.8969(4)	8.6982(2)	41.2123(19)	8.6833(2)	17.1922(6)
b [Å]	8.6721(1)	5.1581(1)	20.8981(4)	20.7682(6)	21.0160(4)	20.9911(8)
c [Å]	16.6501(2)	39.7013(7)	21.6926(5)	51.7215(12)	21.6241(5)	22.4727(12)
β [°]	–	100.871(2)	110.687(2)	104.778(3)	110.459(2)	115.394(3)
V [Å^3]	2113.17(6)	5208.08(16)	3688.95(14)	42804(3)	3697.23(14)	7326.4(5)
Z	8	16	4	48	4	8
D_{calc} [gcm^{-3}]			1.484	1.535		
μ [mm^{-1}]	0.770	0.797	4.086	4.226	5.250	5.299
Measured reflections	5238	10272	14746	183463	16029	32771
Independent reflections	2082	5143	7227	134249	7277	14397
Observed reflections [$I > 4\sigma(I)$]	1921	4610	5856	80553		
R_{int}	0.017	0.017	0.021	0.037	0.029	0.033
R_1, $I > 2\sigma(I)$[a]	0.033	0.036	0.077	0.084	0.077	0.079
wR_2, all data[b]	0.084	0.091	0.227	0.269	0.211	0.223
GoF	1.050	1.089	1.156	1.058		
Flack parameter	–	–	–	0.0(4)	–	–

[a]$R = \Sigma[|F_o| - |F_c|] / \Sigma|F_o|$ [b]$wR = [\Sigma w(F_o^2 - F_c^2) / \Sigma wF_o^4]^{1/2}$

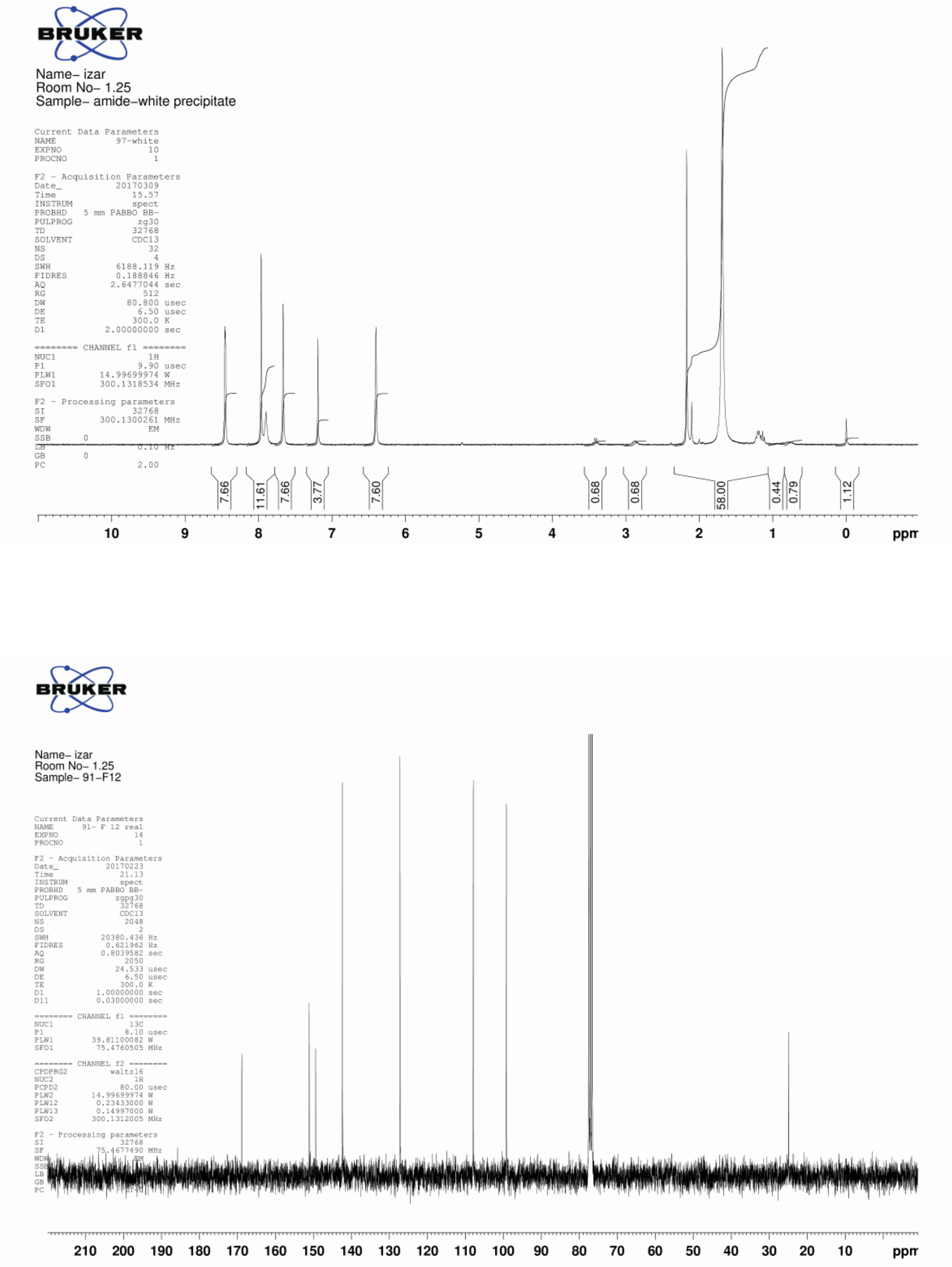

Figure S1. ^{1}H (top) and ^{13}C (bottom) NMR spectra of *L* ($CDCl_3$).

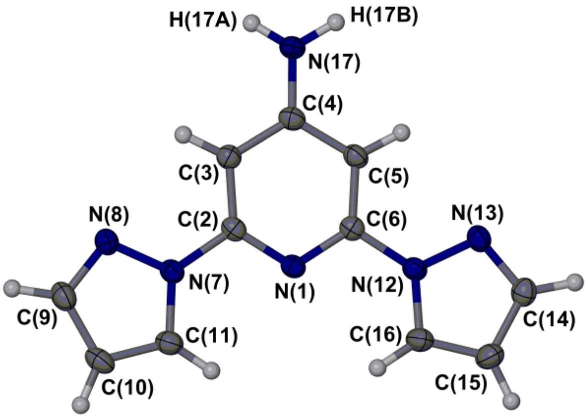

Figure S2. Asymmetric unit of bpp^{NH2}, showing the atom numbering scheme. Displacement ellipsoids are at the 50 % probability level. Color code: C, dark gray; H, pale gray; N, blue.

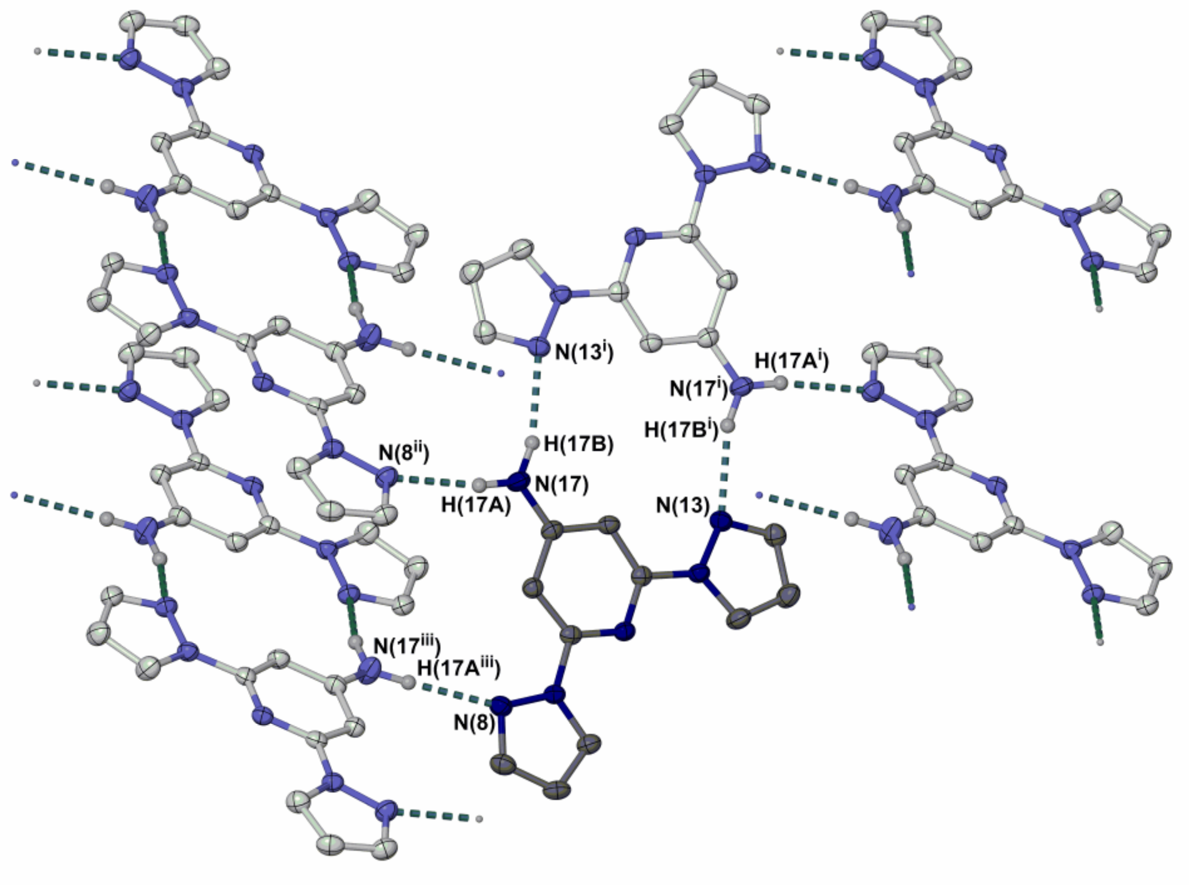

Figure S3. The puckered 2D hydrogen bond network in crystalline bpp^{NH2}. Displacement ellipsoids are at the 50 % probability level, and C-bound H atoms are omitted for clarity. The view is along the crystallographic [110] vector, with the *c* axis horizontal. The molecule in the asymmetric unit has dark coloration, while the symmetry-related molecules have pale color.

Symmetry codes (i) 1–*x*, 2–*y*, 1–*z*; (ii) 1–*x*, $^{1}/_{2}$+*y*, $^{1}/_{2}$–*z*; (iii) 1–*x*, –$^{1}/_{2}$+*y*, $^{1}/_{2}$–*z*. Color code: C, dark gray or white; H, pale gray; N, pale or dark blue.

The molecules associate into centrosymmetric dimers through the N(17)–H(17B)…N(13^{i}) hydrogen bond, which are then linked by the N(17)–H(17A)…N(8^{ii}) interaction into puckered 2D sheets parallel to (100).

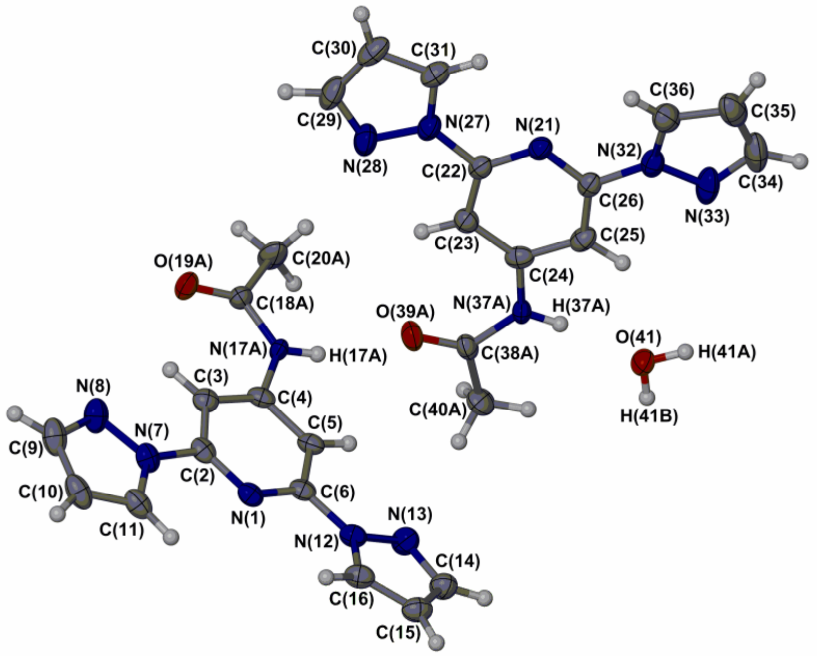

Figure S4. Asymmetric unit of $L\cdot¼H_2O$, showing the atom numbering scheme (the water molecule O(41) is half-occupied). Only one orientation of the disordered acetamido groups is shown, and displacement ellipsoids are at the 50 % probability level. Color code: C, dark gray; H, pale gray; N, blue; O, red.

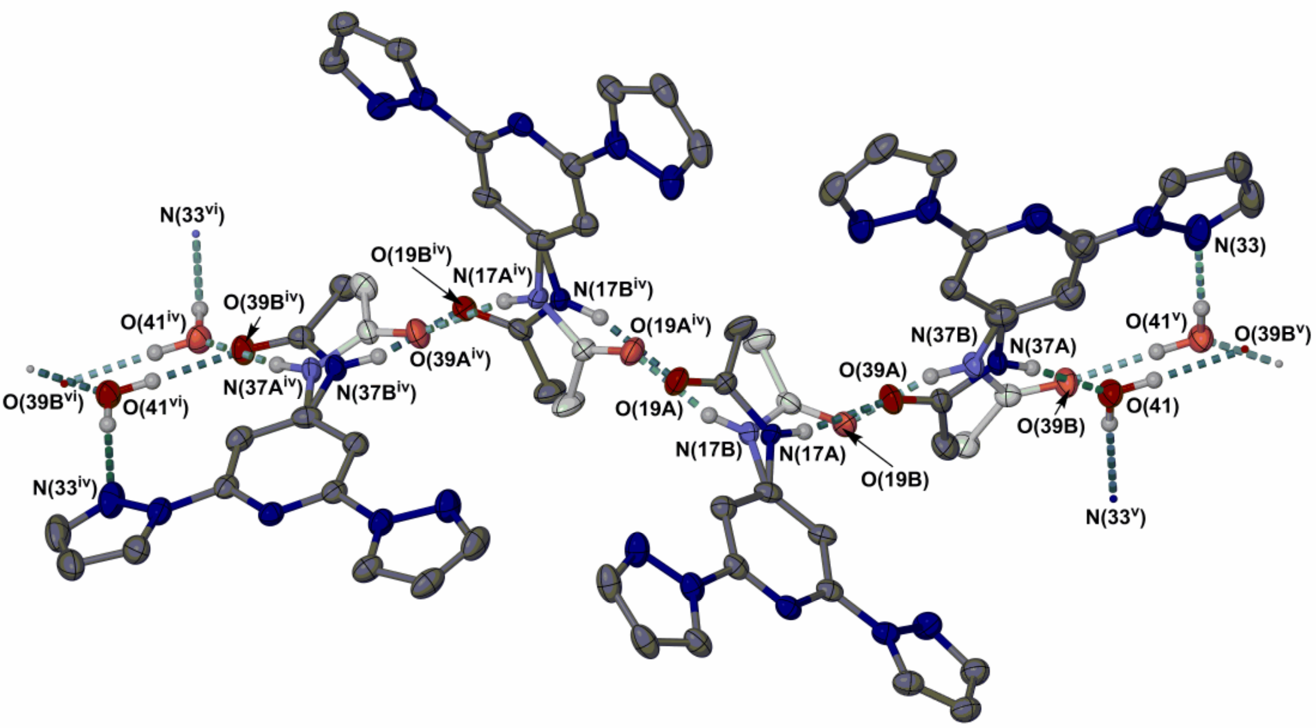

Figure S5. A hydrogen-bonded $[L_4(H_2O)]$ tetrad in $L\cdot{}^1/_4H_2O$. The two half-occupied disorder orientations of the hydrogen-bond chains are shown in pale and dark coloration [water molecule O(41) and its symmetry-equivalents are also half-occupied]. Displacement ellipsoids are at the 50 % probability level, and C-bound H atoms are omitted for clarity. The view is along the crystallographic [010] vector, with the *c* axis horizontal.

Symmetry codes (iv) 1–*x*, *y*, $^3/_2$–*z*; (v) 1–*x*, 3–*y*, 1–*z*; (vi) *x*, 3–*y*, $^1/_2$+*z*. Color code: C, dark gray or white; H, pale gray; N, pale or dark blue; O, pale or dark red.

Table S2 Hydrogen bond parameters for the organic ligand structures in this work [Å, °]. See Figures S2-S5 for the atom numbering schemes. Symmetry codes: (i) 1–*x*, 2–*y*, 1–*z*; (ii) 1–*x*, $^1/_2$+*y*, $^1/_2$–*z*; (iv) 1–*x*, *y*, $^3/_2$–*z*; (v) 1–*x*, 3–*y*, 1–*z*.

	D–H	H…A	D…A	D–H…A
bpp^{NH2}				
N(17A)–H(17A)…N(8^{i})	0.914(16)	2.195(17)	3.0988(15)	169.6(13)
N(17B)–H(17B)…N(13^{ii})	0.955(18)	2.153(18)	3.0643(14)	158.9(14)
$L\cdot{}^1/_4H_2O$				
N(17A)–H(17A)…O(39A)	0.88	2.02	2.862(5)	159.5
N(17B)–H(17B)…O($19A^{iv}$)	0.88	2.05	2.911(5)	164.5
N(37A)–H(37A)…O(41)	0.88	1.93	2.800(5)	170.5
N(37B)–H(37B)…O(19B)	0.88	2.03	2.887(4)	164.3
O(41)–H(41A)…O($39B^{v}$)	0.906(19)	1.91(2)	2.814(3)	176(3)
O(41)–H(41B)…N(33^{v})	0.905(19)	2.252(19)	3.156(2)	177(3)

Definitions of the Structural Parameters in Tables S3 and S6

V_{Oh} is the volume (in Å^3) of the FeN_6 coordination octahedron in the complex molecule.[8] Σ and Θ are defined as follows:

$$\Sigma = \sum_{i=1}^{12} |90 - \beta_i| \qquad \Theta = \sum_{j=1}^{24} |60 - \gamma_j|$$

where β_i are the twelve *cis*-N–Fe–N angles about the iron atom and γ_i are the 24 unique N–Fe–N angles measured on the projection of two triangular faces of the octahedron along their common pseudo-threefold axis (Scheme S1). Σ is a general measure of the deviation of a metal ion from an ideal octahedral geometry, while Θ more specifically indicates its distortion towards a trigonal prismatic structure. A perfectly octahedral complex gives $\Sigma = \Theta = 0$.[8,9]

Because the high-spin state of a complex has a much more plastic structure than the low-spin, this is reflected in Σ and Θ which are usually much larger in the high-spin state.

The crystallographic spin states of individual molecules in this work were assigned from the following criteria, based on literature precedent:[10,11]

High-spin: $V_{Oh} > 11.5$ Å^3; $\Sigma > 145$ °; $\Theta > 450$ °.
Low-spin: $V_{Oh} < 10.0$ Å^3; $\Sigma < 100$ °; $\Theta < 320$ °.
Mixed spin-state population: intermediate values of V_{Oh}, Σ and Θ.

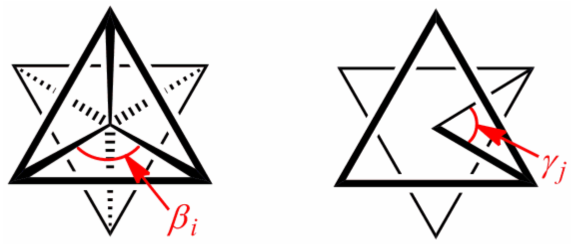

Scheme S1. Angles used in the definitions of the coordination distortion parameters Σ and Θ.

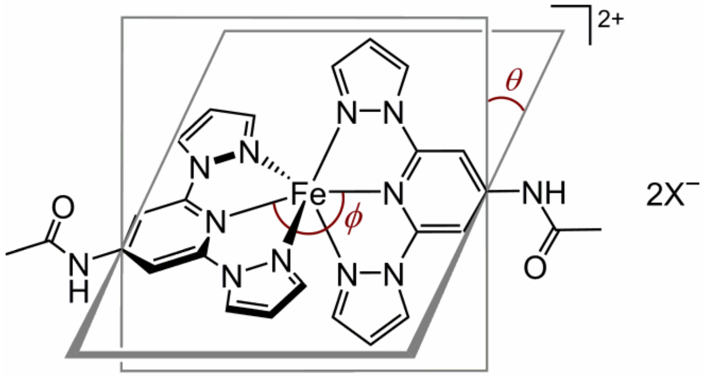

Scheme S2. Definition of the Jahn-Teller distortion parameters θ and ϕ.

These two parameters define the magnitude of an angular Jahn-Teller distortion, that is often observed in high-spin $[Fe(bpp)_2]^{2+}$ (bpp = 2,6-di{pyrazol-1-yl}pyridine) derivatives like $[FeL_2]^{2+}$ ($\theta \leq 90$°, $\phi \leq 180$ °).[11,12] They are also a useful indicator of the molecular geometry, in defining the disposition of the two ligands around the metal ion.

Spin-crossover can be inhibited if θ and ϕ deviate significantly from their ideal values, because the associated rearrangement to a more regular low-spin coordination geometry ($\theta \approx 90$°, $\phi \approx 180$°) cannot be accommodated by a rigid solid lattice.[12,13] Conversely, significant changes in θ and ϕ between the spin states can be associated with greater SCO cooperativity.[14]

Table S3 Selected bond distances and metric parameters for **1[BF$_4$]$_2$**·Me$_2$CO at different temperatures [Å, Å^3, °]. See Figure 2 of the main article for the atom numbering scheme, while definitions of the parameters in the Table are on page 9 of this document (see also the comment on the next page).

T [K]	240	130					
		Molecule A	Molecule B	Molecule C	Molecule D	Molecule E	Molecule F
Spin state[a]	HS	HS	HS	HS	HS	HS	HS
Fe(1)–N(2)	2.121(3)	2.157(6)	2.136(6)	2.147(8)	2.155(6)	2.153(6)	2.153(6)
Fe(1)–N(9)	2.200(3)	2.179(6)	2.216(7)	2.183(7)	2.182(5)	2.184(6)	2.194(7)
Fe(1)–N(14)	2.186(3)	2.176(6)	2.205(7)	2.201(8)	2.190(6)	2.204(6)	2.182(7)
Fe(1)–N(22)	2.116(3)	2.144(6)	2.091(7)	2.113(8)	2.120(6)	2.134(6)	2.111(6)
Fe(1)–N(29)	2.180(4)	2.194(6)	2.164(6)	2.183(7)	2.205(6)	2.171(6)	2.203(6)
Fe(1)–N(34)	2.184(4)	2.208(7)	2.216(6)	2.215(7)	2.214(6)	2.197(6)	2.181(6)
V_{Oh}	12.271(12)	12.147(3)	12.269(3)	12.020(3)	12.259(3)	12.161(3)	12.113(3)
Σ	154.3(4)	163.9(7)	154.1(7)	166(1)	161.3(7)	162.1(7)	160.2(7)
Θ	480	485	485	503	484	489	490
ϕ	169.65(13)	162.4(2)	165.2(2)	161.6(3)	163.6(2)	163.5(2)	164.1(2)
θ	88.50(4)	79.80(6)	84.53(6)	80.02(7)	81.39(6)	81.68(6)	83.59(6)

	Molecule G	Molecule H	Molecule I	Molecule J	Molecule K	Molecule L	Molecule M
Spin state[a]	HS	HS	HS	HS	mixed	mixed	mixed
Fe(1)–N(2)	2.156(6)	2.132(5)	2.137(6)	2.101(5)	2.077(7)	2.081(6)	1.986(7)
Fe(1)–N(9)	2.188(6)	2.203(6)	2.152(6)	2.183(6)	2.122(7)	2.154(6)	2.098(6)
Fe(1)–N(14)	2.195(6)	2.220(6)	2.193(6)	2.193(6)	2.116(7)	2.134(6)	2.101(8)
Fe(1)–N(22)	2.126(6)	2.103(6)	2.104(5)	2.086(7)	2.056(7)	2.073(6)	1.979(8)
Fe(1)–N(29)	2.163(6)	2.164(6)	2.200(6)	2.164(6)	2.151(6)	2.106(7)	2.077(7)
Fe(1)–N(34)	2.192(7)	2.208(5)	2.220(6)	2.199(6)	2.099(7)	2.122(8)	2.050(7)
V_{Oh}	12.262(3)	12.028(3)	12.274(3)	12.187(3)	11.443(3)	11.570(3)	10.811(2)
Σ	157.9(7)	163.7(7)	154.7(7)	147.6(7)	138(1)	143.4(9)	119(1)
Θ	488	500	475	465	431	446	383
ϕ	167.6(2)	161.4(2)	167.0(2)	169.5(2)	170.4(3)	172.4(2)	174.9(2)
θ	86.73(6)	79.89(6)	85.26(6)	89.97(5)	87.64(6)	88.78(6)	86.87(6)

[a]HS = high-spin, LS = low-spin, mixed = a mixed high-spin/low-spin population. The criteria used to assign spin states to each molecule are described on page 9, and at the end of this Table on page 11.

Table S3 continued.

Spin state[a]	Molecule N<br>mixed	Molecule O<br>LS	Molecule P<br>LS	Molecule Q<br>LS	Molecule R<br>LS	Molecule S<br>LS	Molecule T<br>LS
Fe(1)–N(2)	2.022(6)	1.935(6)	1.939(6)	1.902(6)	1.891(5)	1.889(6)	1.922(6)
Fe(1)–N(9)	2.060(6)	2.006(6)	1.990(5)	1.992(6)	2.021(5)	2.038(6)	1.994(6)
Fe(1)–N(14)	2.065(6)	2.006(6)	1.981(6)	1.948(7)	2.010(6)	1.989(6)	1.962(6)
Fe(1)–N(22)	2.018(6)	1.948(6)	1.941(5)	1.924(6)	1.904(5)	1.890(7)	1.928(6)
Fe(1)–N(29)	2.079(7)	1.953(6)	1.998(6)	1.960(7)	1.977(5)	1.991(6)	1.955(7)
Fe(1)–N(34)	2.102(7)	1.978(7)	2.025(6)	1.958(7)	1.974(5)	1.988(7)	1.975(6)
V_{Oh}	10.917(2)	9.833(1)	9.931(2)	9.518(2)	9.719(2)	9.750(2)	9.616(2)
Σ	121.2(8)	94.9(9)	97.1(7)	89(1)	93.6(7)	91(1)	92.6(9)
Θ	383	305	314	288	306	299	299
ϕ	172.6(2)	176.3(2)	175.0(2)	175.5(3)	177.2(2)	177.2(3)	174.9(2)
θ	88.87(6)	87.15(6)	89.59(5)	87.42(6)	86.43(5)	87.05(5)	88.01(5)

Spin state[a]	Molecule U<br>LS	Molecule V<br>LS	Molecule W<br>LS	Molecule X<br>LS
Fe(1)–N(2)	1.889(6)	1.915(6)	1.909(5)	1.881(6)
Fe(1)–N(9)	1.990(5)	2.031(5)	2.027(5)	2.035(6)
Fe(1)–N(14)	1.984(6)	1.984(6)	1.980(5)	1.994(6)
Fe(1)–N(22)	1.927(6)	1.927(5)	1.883(5)	1.913(6)
Fe(1)–N(29)	1.960(6)	2.043(5)	1.997(5)	1.971(6)
Fe(1)–N(34)	1.938(7)	1.965(5)	1.956(6)	1.981(6)
V_{Oh}	9.534(2)	9.859(2)	9.653(2)	9.710(2)
Σ	88.4(9)	99.9(7)	91.5(7)	93.6(8)
Θ	287	326	301	307
ϕ	177.2(3)	173.8(2)	175.9(2)	177.4(3)
θ	87.04(6)	89.70(5)	88.24(5)	86.73(5)

[a]HS = high-spin, LS = low-spin, mixed = a mixed high-spin/low-spin population. The criteria used to assign spin states to each molecule are described on page 9.

Some spin state assignments in the Table are not clear cut. For example, Σ and Θ for molecules J-L all lie near the high-spin/mixed-spin boundary, but molecule J was assigned as high-spin because of its higher V_{Oh} value. Similarly, V_{Oh} for molecule V looks low-spin, but its Σ and Θ values are slightly higher than expected for a purely low-spin molecule.

The mixed-spin molecules K and L have a majority high-spin population at this temperature. The other mixed-spin molecules M and N are closer to a 1:1 high:low-spin ratio.

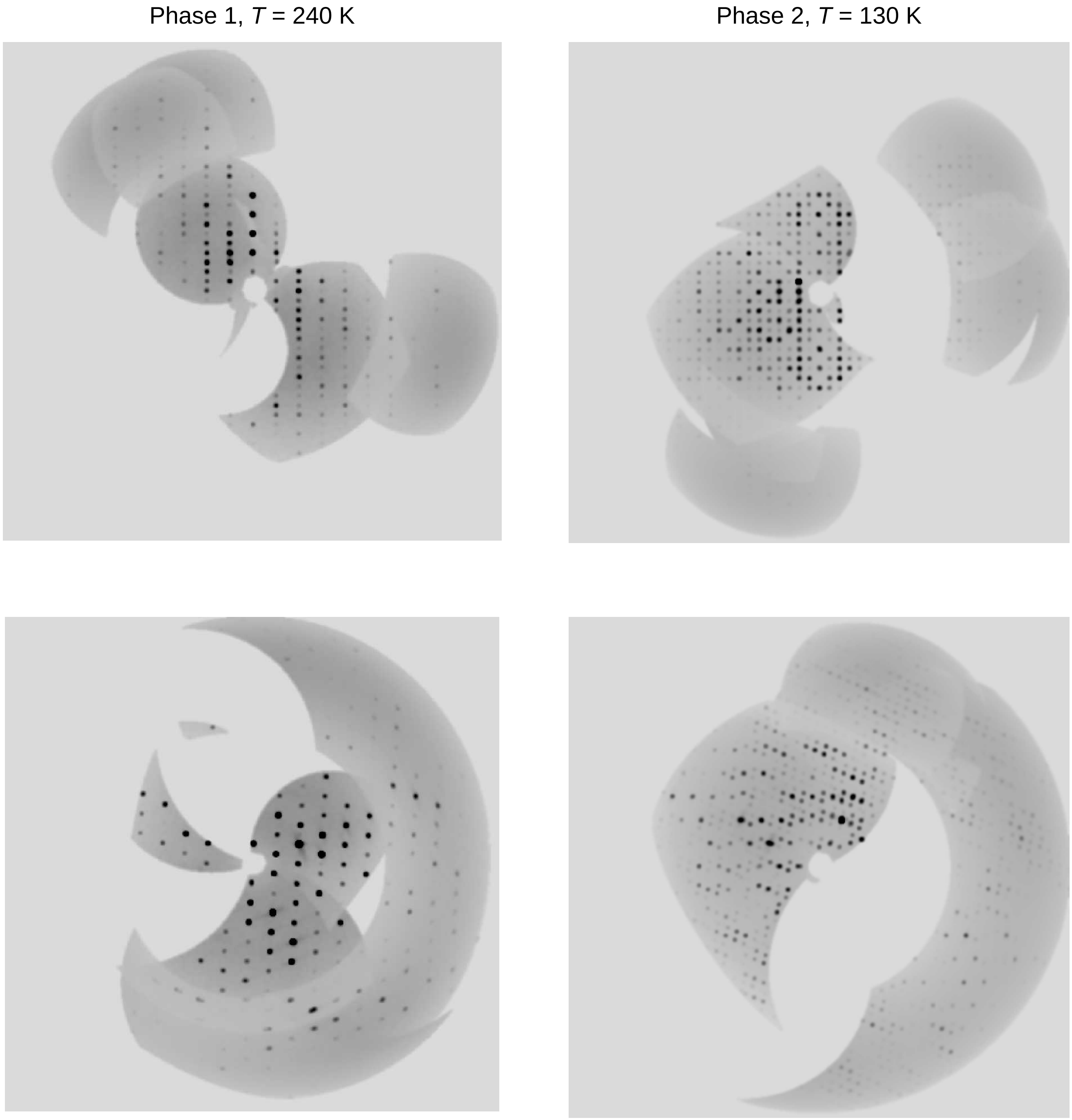

Figure S6. Diffraction images from **1[BF$_4$]$_2$**·Me$_2$CO in the *hk*0 (top) and *h*0*l* (bottom) zones, showing the large number of additional diffraction spots afforded by phase 2.

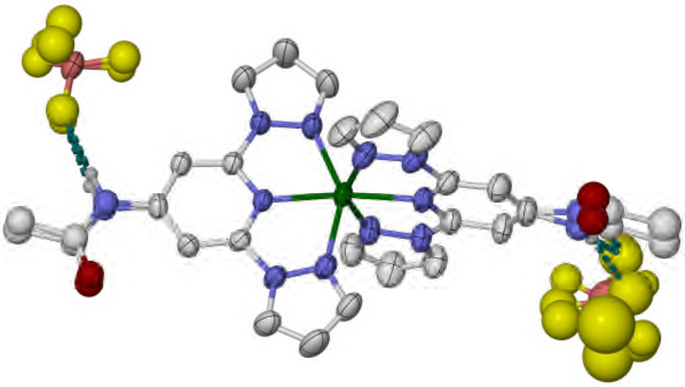

Figure S7. The $[FeL_2][BF_4]_2$ assembly in phase 1 of **$1[BF_4]_2$**$\cdot Me_2CO$ at 240 K. Displacement ellipsoids are at the 50 % probability level, and C-bound H atoms have been omitted for clarity. The view is the same as in Fig. S8, to enable comparison.

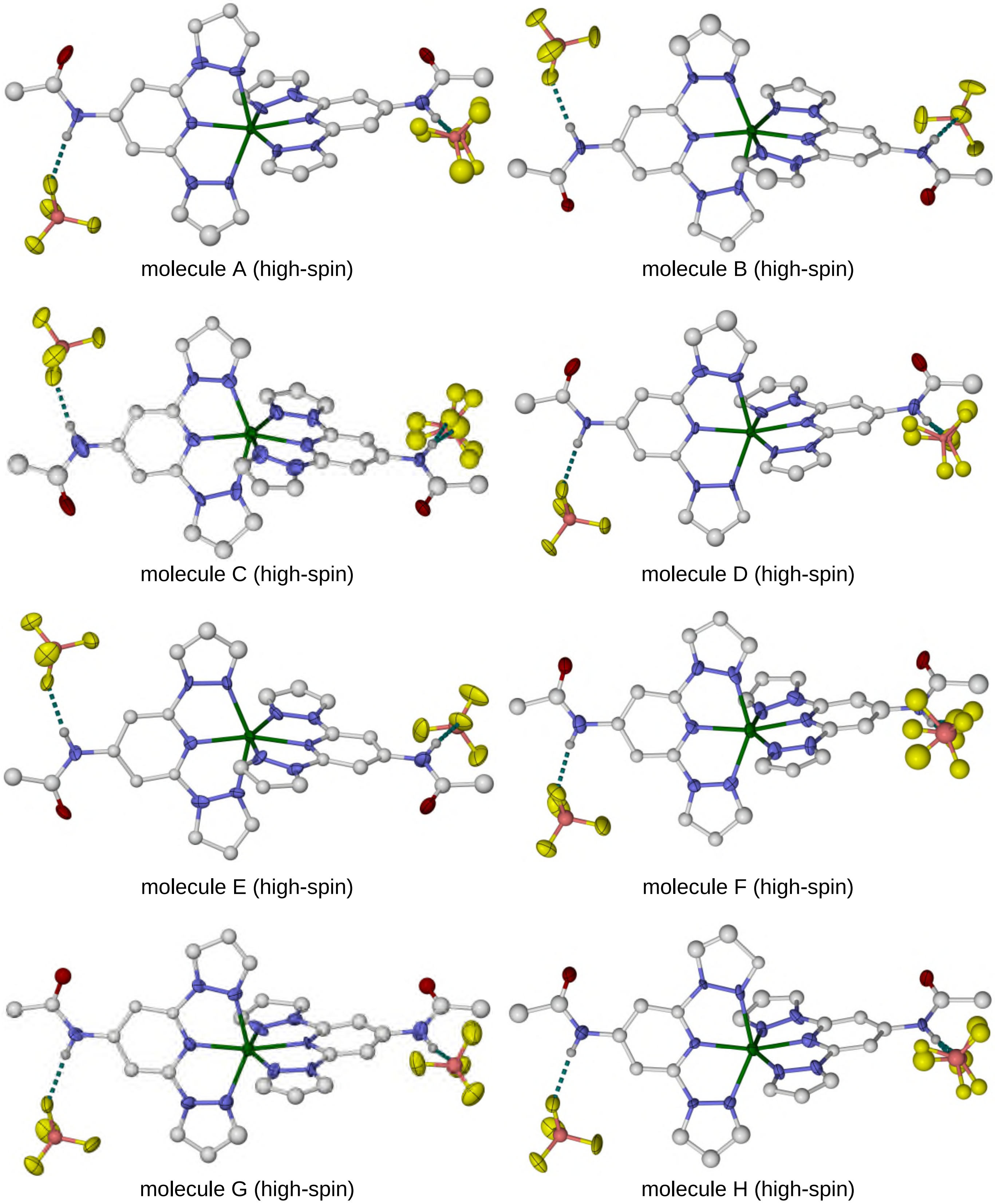

Figure S8. The twenty-four unique [FeL_2][BF_4]$_2$ moieties in phase 2 of **1[BF_4]$_2$**·Me_2CO at 130 K. Displacement ellipsoids are at the 50 % probability level, and C-bound H atoms have been omitted for clarity. All orientations of disordered BF_4^- ions are shown. The view in each case is parallel to the [001] crystallographic vector with the unit cell *b* axis horizontal.

Color code: C, white; H, pale gray; B, pink; F, yellow; Fe, green; N, blue; O, red.

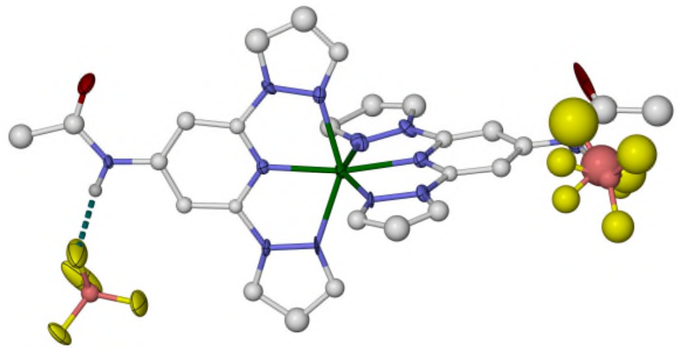

molecule I (high-spin)

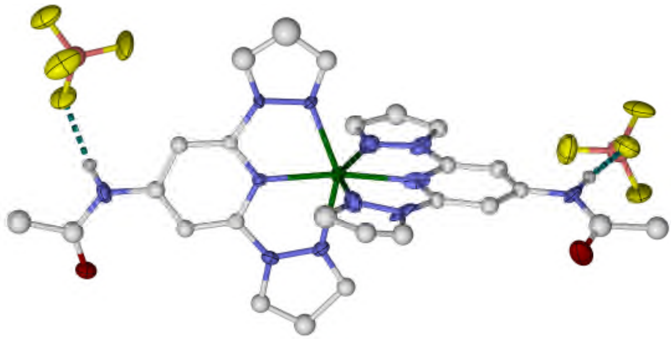

molecule J (high-spin)

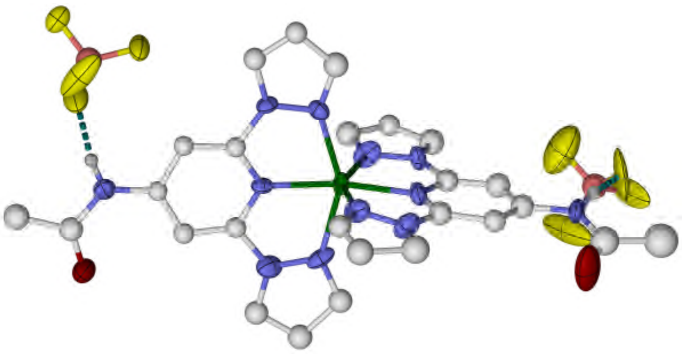

molecule K (mixed-spin)

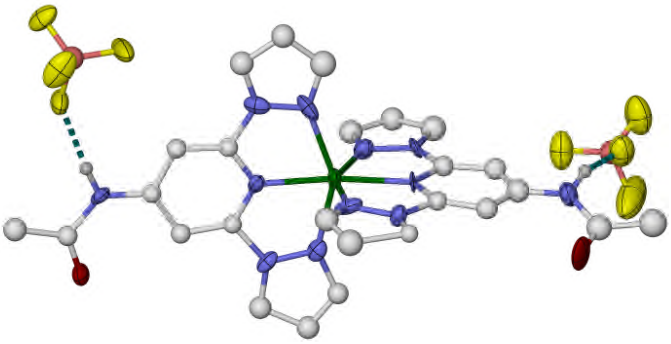

molecule L (mixed-spin)

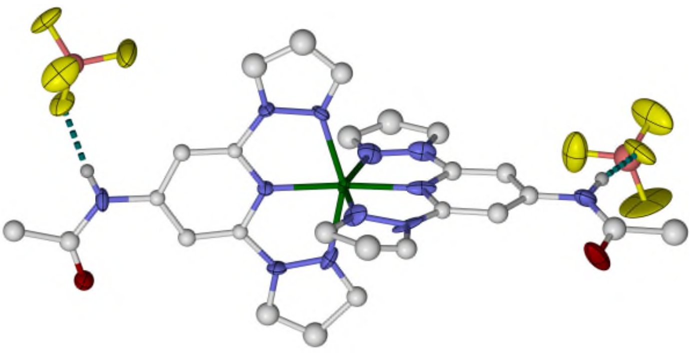

molecule M (mixed-spin)

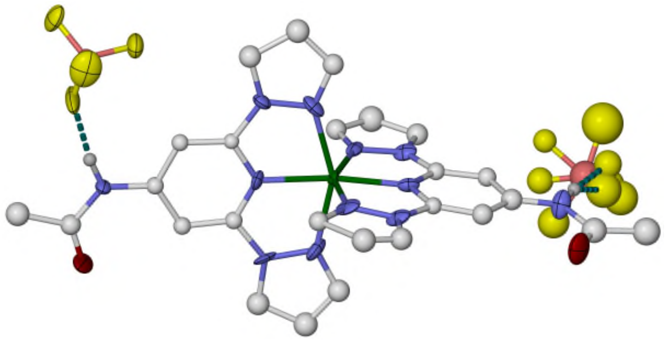

molecule N (mixed-spin)

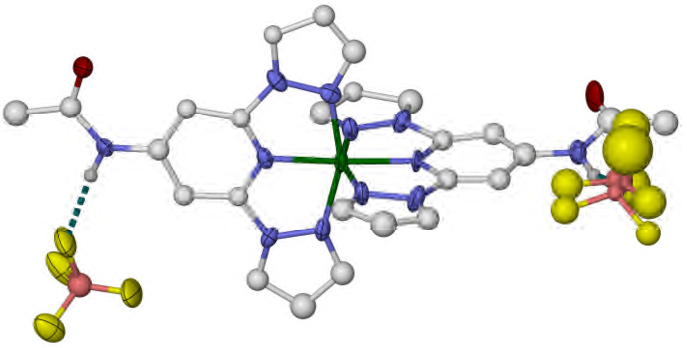

molecule O (low-spin)

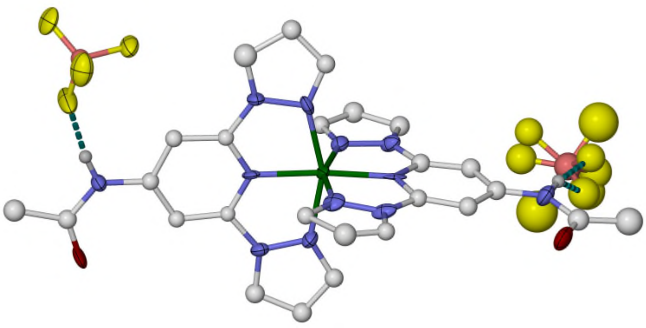

molecule P (low-spin)

Figure S8 (continued).

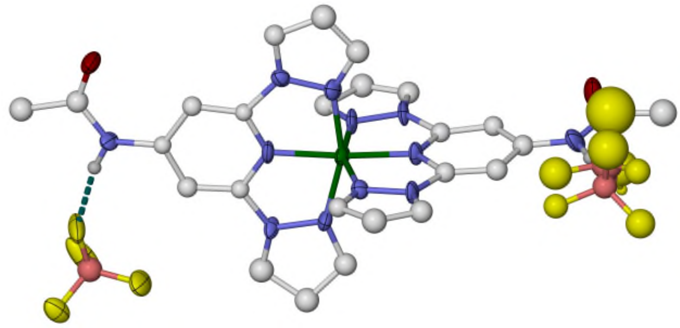

molecule Q (low-spin)

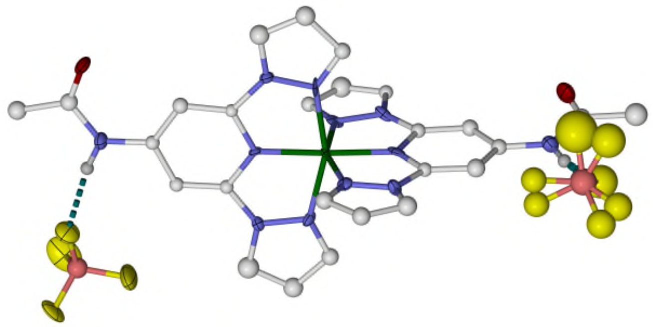

molecule R (low-spin)

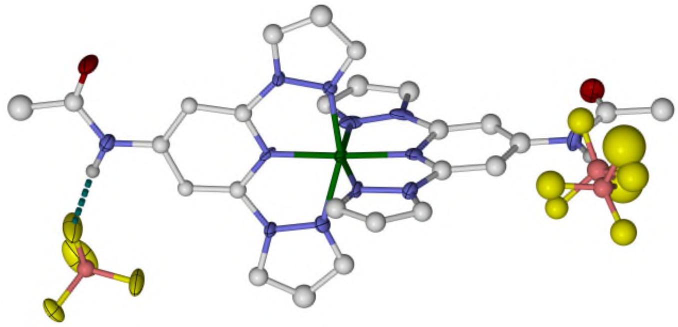

molecule S (low-spin)

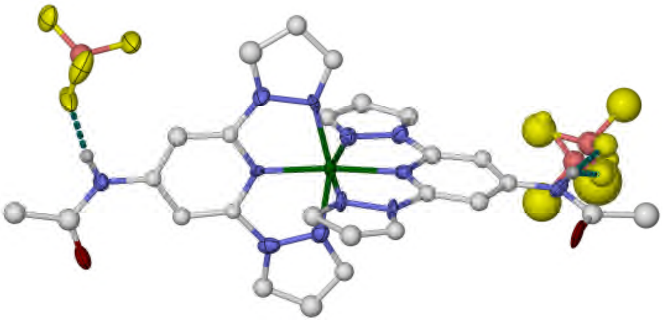

molecule T (low-spin)

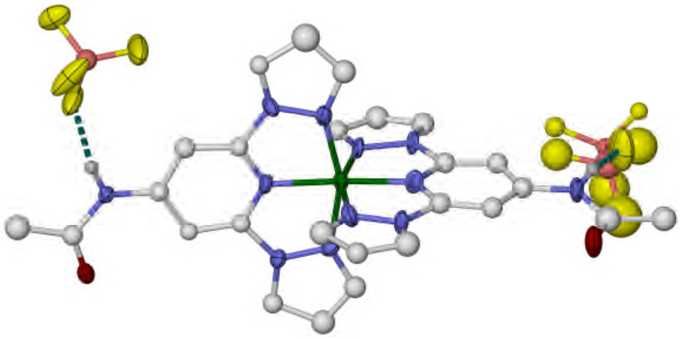

molecule U (low-spin)

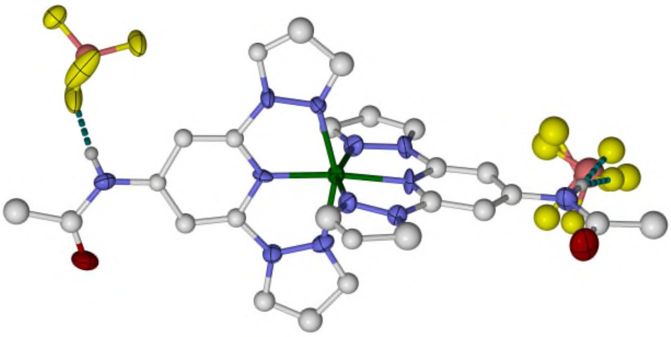

molecule V (low-spin)

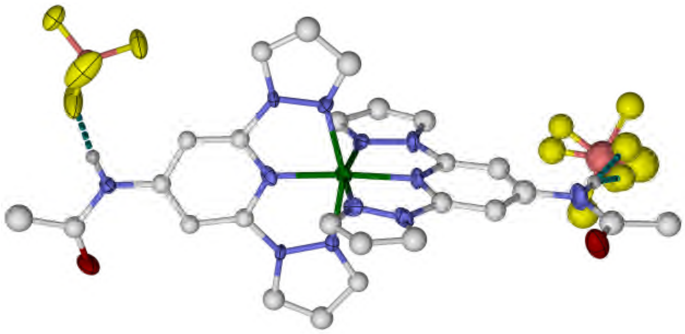

molecule W (low-spin)

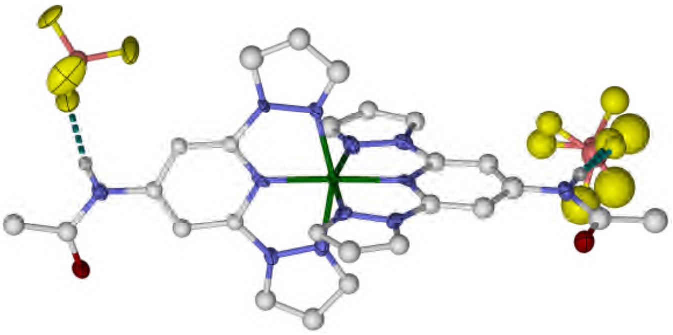

molecule X (low-spin)

Figure S8 (continued).

Table S4 Hydrogen bond parameters for **1[BF$_4$]$_2$**·Me$_2$CO [Å, °]. See Figures 2 and S9 for the atom numbering schemes.

	D–H	H…A	D…A	D–H…A
Phase 1, 240 K				
N(18A)–H(18A)…F(48A)/F(48B)	0.87	2.02/2.10	2.867(9)/2.863(10)	164.1/146.0
N(18B)–H(18B)…F(48A)/F(48B)	0.87	2.09/1.95	2.909(10)/2.817(10)	157.7/175.4
N(38A)–H(38A)…F(43A)/F(44B)/F(45C)	0.87	1.79/1.97/2.09	2.651(15)/2.84(2)/2.88(3)	172.3/174.2/150.8
N(38B)–H(38B)…F(43A)/F(44B)	0.87	2.11/2.28	2.892(15)/3.07(2)	149.0/150.7
Phase 2, 130 K				
N(18A)–H(18A)…F(01C)/F(01D)	0.88	1.89/2.19	2.750(8)/3.006(13)	164.9/153.3
N(38A)–H(38A)…F(191)	0.88	1.94	2.811(6)	171.6
N(18B)–H(18B)…F(193)	0.88	2.01	2.880(7)	167.6
N(38B)–H(38B)…F(171)	0.88	1.94	2.815(6)	174.8
N(18C)–H(18C)…F(229)	0.88	1.97	2.844(11)	169.1
N(38C)–H(38C)…F(56C)/F(56D)	0.88	2.05/1.85	2.868(10)/2.704(15)	154.4/161.4
N(18D)–H(18D)…F(43C)/F(43D)	0.88	2.20/1.89	3.017(11)/2.753(10)	154.6/166.2
N(38D)–H(38D)…F(234)	0.88	1.94	2.821(6)	173.8
N(18E)–H(18E)…F(109)	0.88	1.98	2.811(7)	156.0
N(38E)–H(38E)…F(91)	0.88	1.97	2.842(6)	170.7
N(18F)–H(18F)…F(258)	0.88	1.91	2.780(8)	169.7
N(38F)–H(38F)…F(70A)/F(70B)	0.88	1.97/2.15	2.793(8)/2.856(19)	155.7/136.9
N(18G)–H(18G)…F(151)	0.88	2.01	2.813(7)	151.4
N(38G)–H(38G)…F(129)	0.88	1.97	2.836(6)	169.8
N(18H)–H(18H)…F(255)	0.88	2.03	2.899(7)	168.3
N(38H)–H(38H)…F(39C)/F(39D)	0.88	2.11/1.86	2.955(11)/2.731(10)	159.6/168.3
N(18I)–H(18I)…F(61A)/F(61B)	0.88	2.10/1.97	2.935(14)/2.841(16)	158.6/168.9
N(38I)–H(38I)…F(186)	0.88	1.96	2.828(6)	167.7
N(18J)–H(18J)…F(206)	0.88	2.00	2.871(7)	171.6
N(38J)–H(38J)…F(250)	0.88	1.95	2.824(7)	172.1
N(18K)–H(18K)…F(118)	0.88	1.90	2.761(8)	166.5
N(38K)–H(38K)…F(73)	0.88	2.08	2.898(9)	154.3
N(18L)–H(18L)…F(264)	0.88	2.00	2.796(7)	149.6
N(38L)–H(38L)…F(95)	0.88	1.95	2.821(6)	170.2
N(18M)–H(18M)…F(224)	0.88	1.97	2.846(7)	173.0
N(38M)–H(38M)…F(211)	0.88	1.97	2.831(8)	167.2
N(18N)–H(18N)…F(53A)/F(53B)	0.88	2.09/1.88	2.965(9)/2.685(19)	171.2/152.1
N(38N)–H(38N)…F(161)	0.88	1.99	2.854(6)	166.1
N(18O)–H(18O)…F(41A)/F(41B)	0.88	2.09/1.99	2.846(16)/2.808(9)	143.5/154.0
N(38O)–H(38O)…F(124)	0.88	1.95	2.826(6)	172.6
N(18P)–H(18P)…F(46A)/F(46B)	0.88	2.02/2.04	2.893(8)/2.80(2)	171.6/143.7
N(38P)–H(38P)…F(163)	0.88	2.01	2.866(6)	165.5
N(18Q)–H(18Q)…F(81A)/F(81B)	0.88	1.92/1.95	2.754(11)/2.832(9)	157.1/174.7
N(38Q)–H(38Q)…F(116)	0.88	2.00	2.864(6)	166.3
N(18R)–H(18R)…F(213)	0.88	1.98	2.856(5)	170.4
N(38R)–H(38R)…F(81C)/F(80D)	0.88	1.99/1.90	2.862(8)/2.760(16)	170.8/165.8
N(18S)–H(18S)…F(221)	0.88	1.96	2.810(7)	163.0
N(38S)–H(38S)…F(73C)/F(73D)	0.88	2.01/1.96	2.882(10)/2.83(2)	170.9/170.3
N(18T)–H(18T)…F(69C)/F(69D)	0.88	1.92/2.00	2.795(7)/2.798(12)	174.2/150.8
N(38T)–H(38T)…F(146)	0.88	2.03	2.885(6)	165.3
N(18U)–H(18U)…F(66)	0.88	1.97	2.792(7)	155.9
N(38U)–H(38U)…F(136)	0.88	1.99	2.863(6)	169.1

Table S4 continued

	D–H	H…A	D…A	D–H…A
N(18V)–H(18V)…F(104)	0.88	1.92	2.779(6)	164.1
N(38V)–H(38V)…F(98A)/F(98B)	0.88	2.07/2.01	2.910(9)/2.884(11)	158.5/175.9
N(18W)–H(18W)…F(181)	0.88	1.93	2.777(6)	162.4
N(38W)–H(38W)…F(83A)/F(85B)	0.88	1.99/2.07	2.868(8)/2.893(13)	178.6/156.0
N(18X)–H(18X)…F(76)	0.88	1.96	2.824(6)	167.4
N(38X)–H(38X)…F(48A)/F(48B)	0.88	2.10/1.85	2.971(14)/2.721(11)	170.8/171.6

Table S5 Intermolecular *π*…*π* contacts for **1[BF$_4$]$_2$**·Me$_2$CO [Å, °]. See Figures 2 and S9 for the atom numbering schemes. Symmetry code: (vii) –1+*x*, *y*, *z*; (viii) *x*, 1+*y*, *z*; (ix) 1–*x*, ½+*y*, 1–*z*; (x) 1–*x*, –½+*y*, 1–*z*; (xi) 1–*x*, –½+*y*, 2–*z*; (xii) *x*, *y*, 1+z.

	Dihedral angle	Interplanar distance	Horizontal offset
Phase 1, 240 K			
[N(28)–C(32)]…[N(33^{vii})–C(37^{vii})]	3.3(5)	3.57(2)	1.67
Phase 2, 130 K			
[N(8A)–C(12A)]…[N(13G^{viii})–C(17G^{viii})]	2.1(2)	3.42(2)	1.26
[N(28B)–C(32B)]…[N(33M)–C(37M)]	4.3(2)	3.49(2)	1.78
[N(28C)–C(32C)]…[N(33B)–C(37B)]	0.7(3)	3.44(3)	1.18
[N(8D)–C(12D)]…[N(13E^{ix})–C(17E^{ix})]	2.8(3)	3.45(2)	1.12
[N(8E)–C(12E)]… [N(13L)–C(17L)]	4.9(3)	3.47(2)	1.48
[N(28F)–C(32F)]…[N(33H)–C(37H)]	8.9(3)	3.55(2)	0.85
[N(8G)–C(12G)]…[N(13O)–C(17O)]	1.5(2)	3.57(2)	1.83
[N(28H)–C(32H)]…[N(33J^{x})–C(37J^{x})]	5.4(2)	3.42(2)	1.50
[N(8I)–C(12I)]…[N(13A)–C(17A)]	3.6(3)	3.42(2)	1.24
[N(28J)–C(32J)]…[N(33R^{xi})–C(37R^{xi})]	1.7(2)	3.59(2)	1.94
[N(28K)–C(32K)]…[N(33C)–C(37C)]	13.1(4)	3.60(2)	0.90
[N(8L)–C(12L)]…[N(13U)–C(17U)]	3.8(2)	3.62(2)	2.05
[N(28M)–C(32M)]…[N(33X^{xii})–C(37X^{xii})]	5.4(2)	3.69(2)	2.07
[N(8N)–C(12N)]…[N(13D^{xi})–C(17D^{xi})]	4.9(3)	3.49(2)	1.42
[N(8O)–C(12O)]…[N(13Q)–C(17Q)]	7.2(2)	3.63(3)	2.38
[N(8P)–C(12P)]…[N(13I^{xi})–C(17I^{xi})]	5.6(2)	3.52(2)	1.76
[N(8Q)–C(12Q)]…[N(13P^{x})–C(17P^{x})]	4.9(2)	3.57(3)	2.40
[N(28R)–C(32R)]…[N(33S^{viii})–C(37S^{viii})]	4.7(2)	3.69(3)	2.34
[N(28S)–C(32S)]…[N(33V^{x})–C(37V^{x})]	6.4(2)	3.63(3)	2.22
[N(8T)–C(12T)]…[N(13N)–C(17N)]	3.8(2)	3.51(3)	2.23
[N(8U)–C(12U)]…[N(13T)–C(17T)]	6.2(2)	3.62(3)	2.49
[N(28V)–C(32V)]…[N(33F^{ix})–C(37F^{ix})]	13.7(3)	3.64(2)	1.28
[N(28W)–C(32W)]…[N(33K)–C(37K)]	8.9(3)	3.67(3)	1.78
[N(28X)–C(32X)]…[N(33W)–C(37W)]	6.0(2)	3.66(3)	2.40

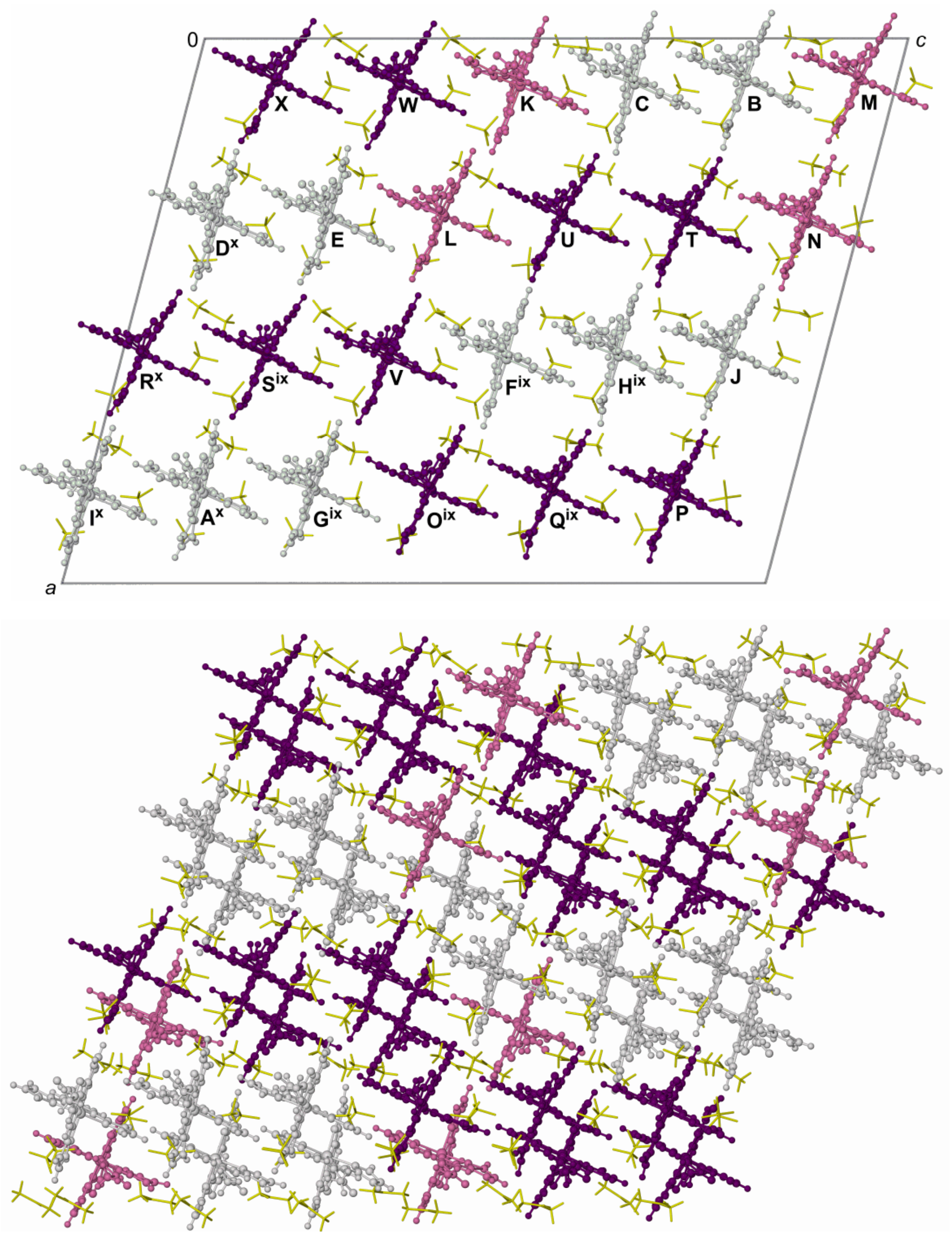

Figure S9. The asymmetric unit (top) and the full unit cell contents (bottom) of phase 2 of **1[BF$_4$]$_2$**·Me$_2$CO. The unit cell comprises two offset layers of molecules related by a 2_1 screw axis, which is perpendicular to the plane of the view. The top view is the same as in Figure 3 of the main article, but with added symmetry codes showing the relationship of each molecule in the view to the crystallographic refinement model.

The view is parallel to the [010] crystal vector, with the unit cell c axis horizontal. High-spin cations are colored white, low-spin cations are dark purple and cations with a mixed high/low-spin population are pale purple; anions and solvent (yellow) are de-emphasized for clarity.

Symmetry codes: (ix) 1–*x*, ½+*y*, 1–*z*; (x) 1–*x*, –½+*y*, 1–*z*.

Table S6 Selected bond distances and angular parameters for **1[ClO$_4$]$_2$**·Me$_2$CO at different temperatures [Å, Å^3, °]. The atom numbering scheme is the same as in Figure 2 of the main article, while definitions of the parameters in the Table are on page 9 of this document.

T [K]	170(2)	120(2)<br>Molecule A	Molecule B
Spin state[a]	HS	HS	HS
Fe(1)–N(2)	2.125(3)	2.120(3)	2.121(3)
Fe(1)–N(9)	2.193(3)	2.199(3)	2.176(4)
Fe(1)–N(14)	2.184(4)	2.176(3)	2.192(4)
Fe(1)–N(22)	2.115(3)	2.114(3)	2.128(3)
Fe(1)–N(29)	2.177(4)	2.174(4)	2.198(3)
Fe(1)–N(34)	2.182(4)	2.180(3)	2.183(3)
V_{Oh}	12.200(2)	12.263(3)	12.148(3)
Σ	155.2(5)	153.5(4)	155.3(4)
Θ	483	476	485
ϕ	168.48(13)	172.57(13)	165.45(12)
θ	87.94(4)	87.50(4)	82.85(4)

[a]HS = high-spin. The criteria used to assign spin states to each molecule are described on page 9.

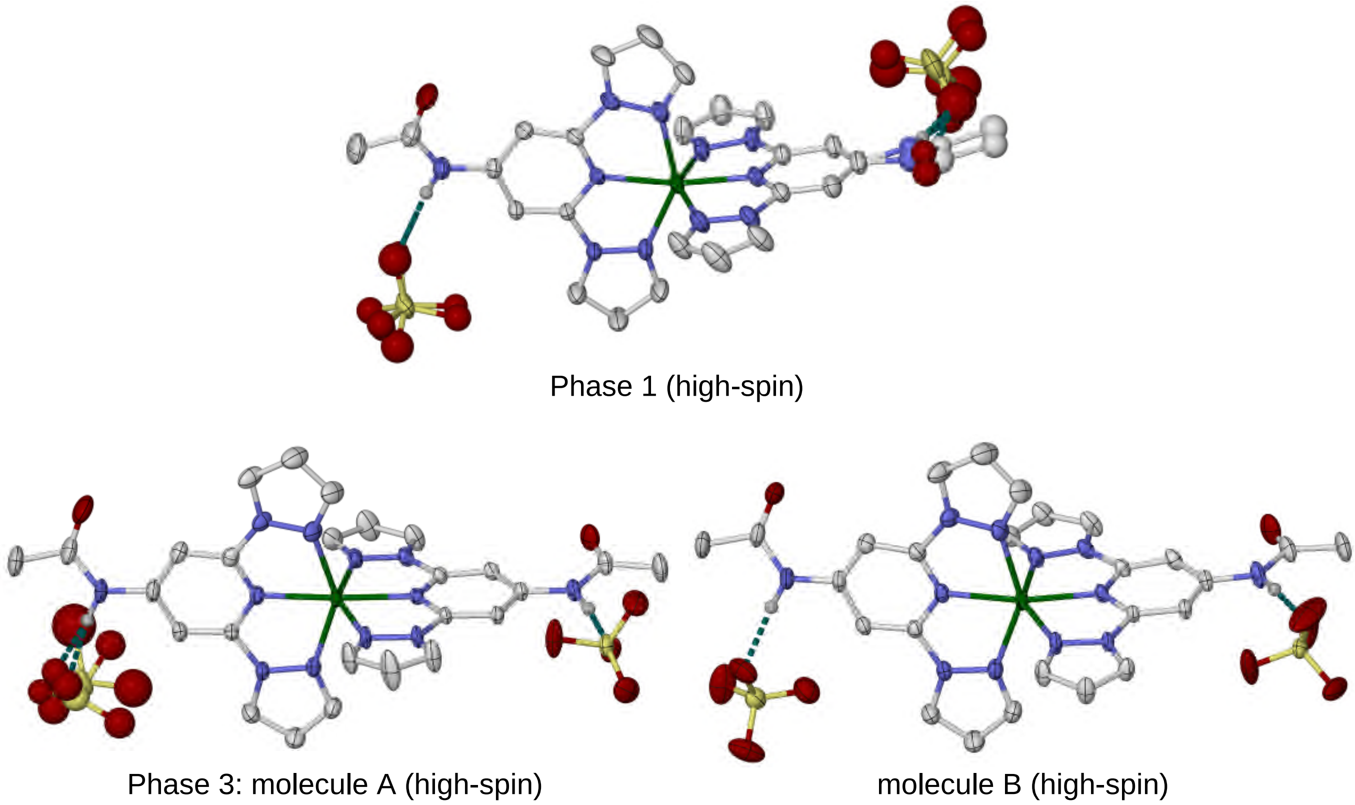

Figure S10. The unique [Fe*L*$_2$][ClO$_4$]$_2$ moieties in both phases of **1[ClO$_4$]$_2$**·Me$_2$CO. Displacement ellipsoids are at the 50 % probability level, and C-bound H atoms have been omitted for clarity. All orientations of disordered BF_4^- ions are shown. The views are the same as in Figs. S7 and S8.

Color code: C, white; H, pale gray; Cl, pale yellow; Fe, green; N, blue; O, red.

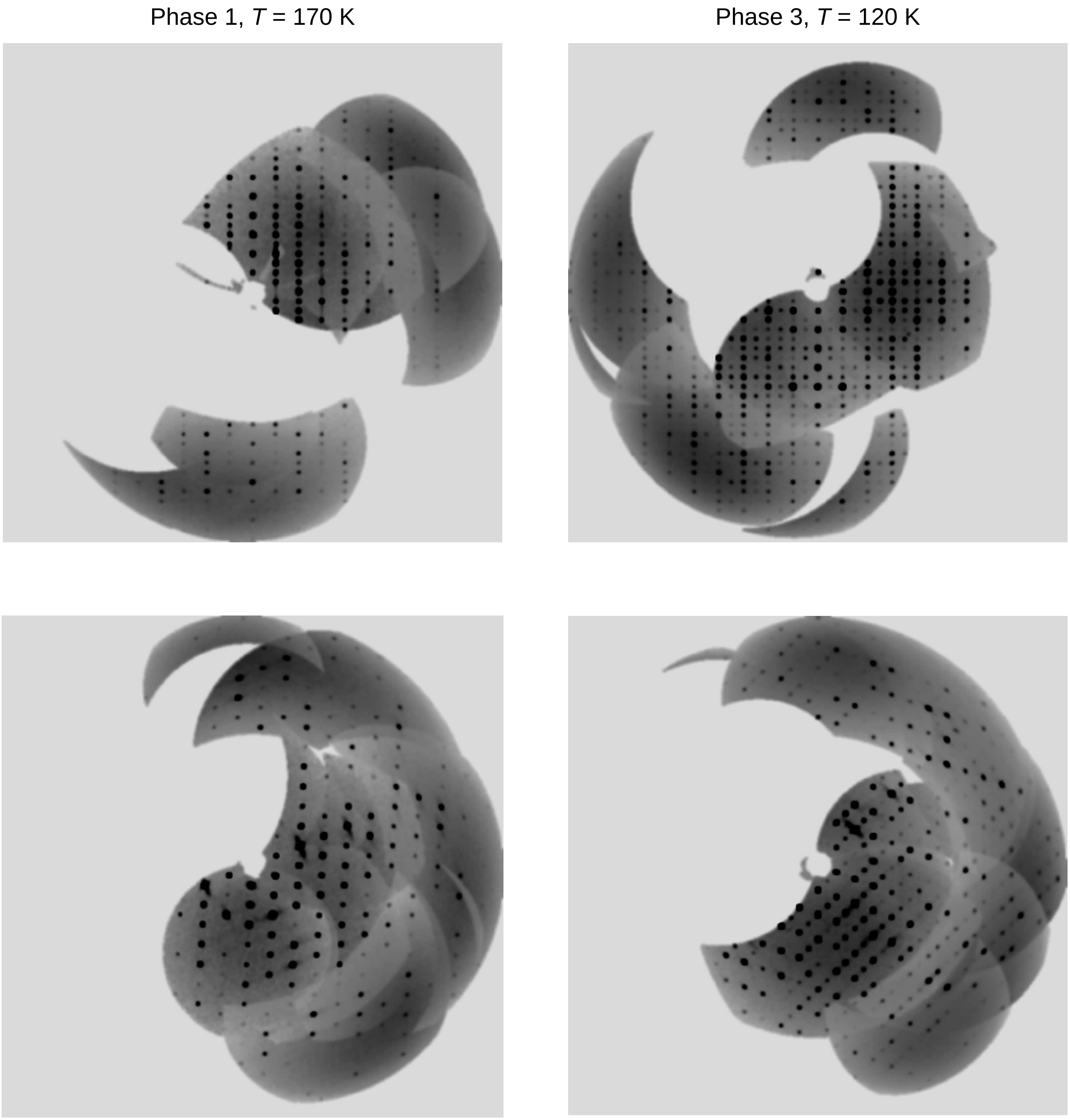

Figure S11. Diffraction images from **1[ClO$_4$]$_2$**·Me$_2$CO in the *hk*0 (top) and *h*0*l* (bottom) zones, showing the doubling of the unit cell *a* parameter in phase 3.

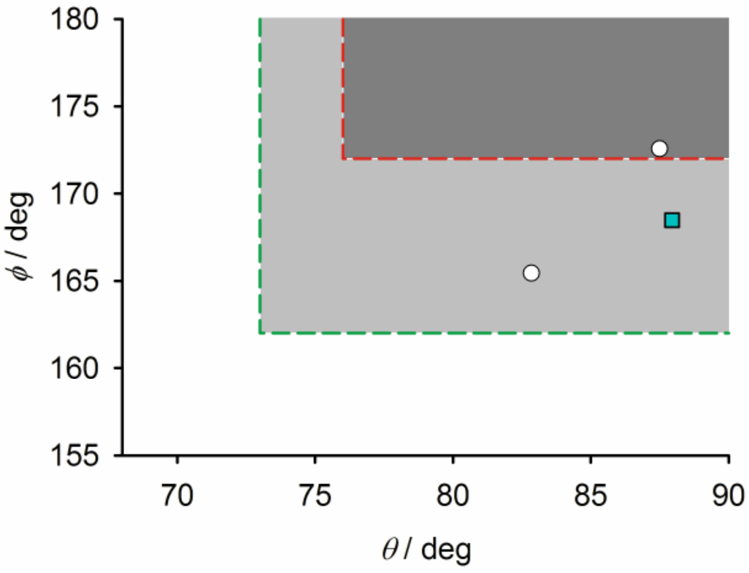

Figure S12. Molecular geometries of the cations in **1[ClO$_4$]$_2$**·Me$_2$CO. The cyan square is the phase 1 cation, while the phase 3 molecules are white circles. See page 9 above for definitions of ϕ and θ. Equivalent data for **1[BF$_4$]$_2$**·Me$_2$CO are plotted in Figure 4 of the main article.

Table S7 Hydrogen bond parameters for **1[ClO$_4$]$_2$**·Me$_2$CO [Å, °]. See Figures 2 and S10 for the atom numbering schemes.

	D–H	H…A	D…A	D–H…A
Phase 1, 170 K				
N(18)–H(18)…O(51A)/O(51B)	0.88	2.05/2.00	2.927(6)/2.855(10)	172.6/162.6
N(38A)–H(38A)…O(43A)/O(43B)	0.88	1.95/1.90	2.816(17)/2.777(13)	167.7/177.1
N(38B)–H(38B)…O(43A)/O(43B)	0.88	2.24/2.10	2.993(18)/2.914(14)	143.4/153.5
Phase 3, 120 K				
N(18A)–H(18A)…O(51)	0.88	2.01	2.884(5)	169.5
N(38A)–H(38A)…O(44A)/O(44B)	0.88	2.16/1.99	3.001(10)/2.824(14)	158.8/156.6
N(18B)–H(18B)…O(61)	0.88	1.98	2.847(5)	168.4
N(38B)–H(38B)…O(53)	0.88	2.04	2.918(5)	177.3

Table S8 Intermolecular $\pi \ldots \pi$ contacts for **1[ClO$_4$]$_2$**·Me$_2$CO [Å, °]. See Figures 2 and S10 for the atom numbering schemes. Symmetry code: (vii) –1+*x*, *y*, *z*. (xiii) 1+*x*, *y*, *z*.

	Dihedral angle	Interplanar distance	Horizontal offset
Phase 1, 170 K			
[N(28)–C(32)]…[N(33^{vii})–C(37^{vii})]	2.5(4)	3.57(2)	1.69
Phase 3, 120 K			
[N(28A)–C(32A)]…[N(8B)–C(12B)]	3.7(2)	3.59(2)	1.46
[N(33A)–C(37A)]…[N(13B^{xiii})–C(17B^{xiii})]	5.8(2)	3.48(2)	1.67

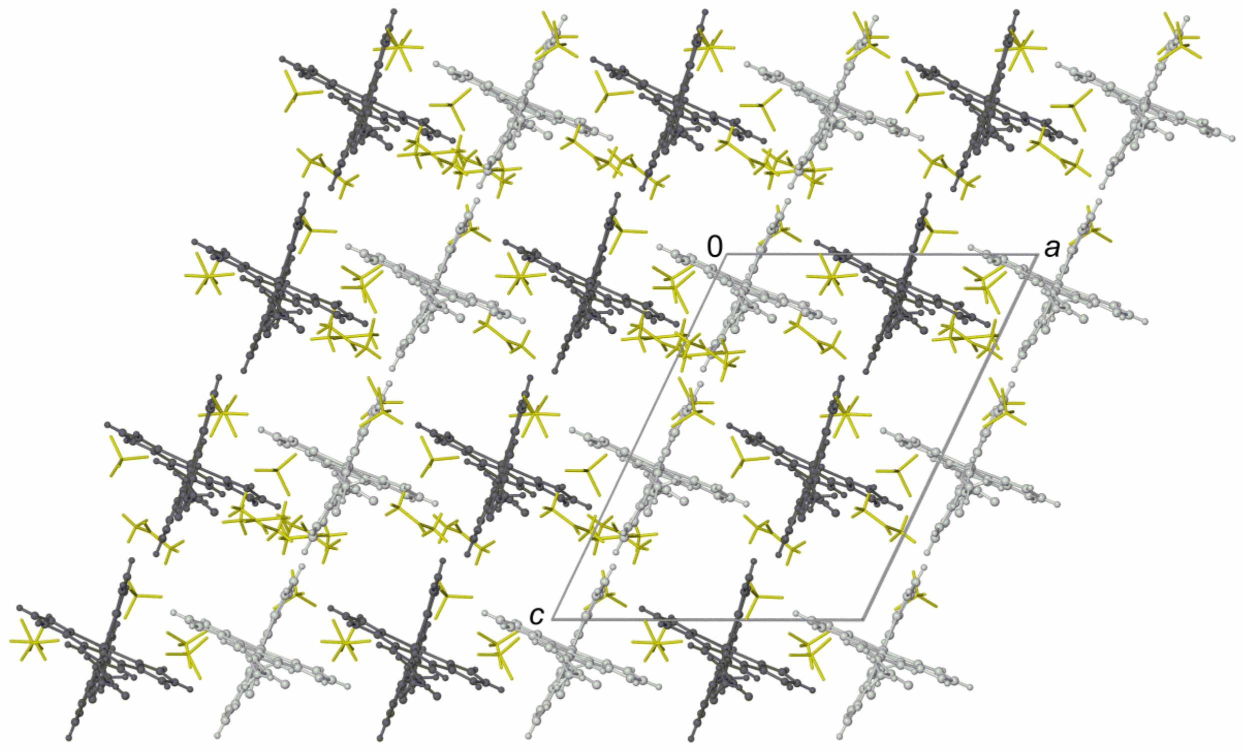

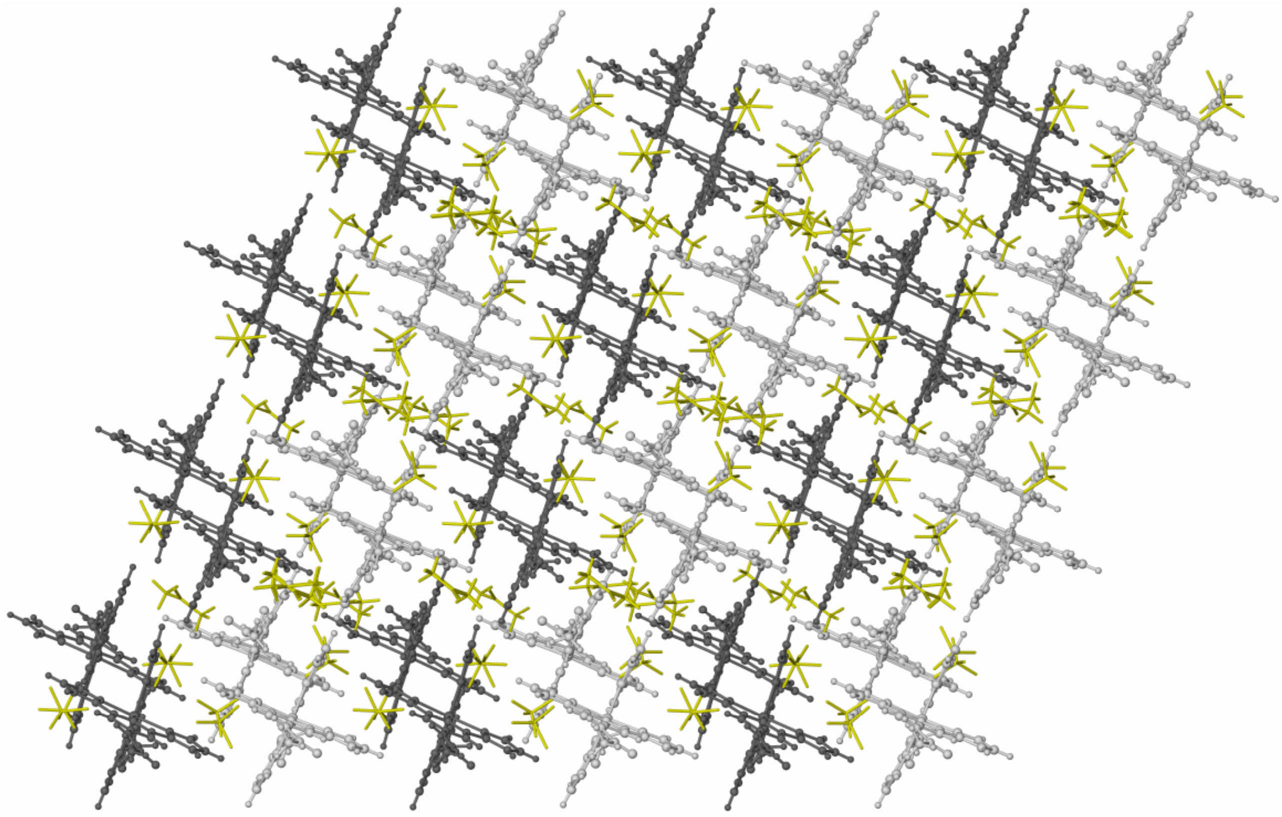

Figure S13. One layer of molecules (top) and a full packing diagram (bottom) of **1[ClO$_4$]$_2$**·Me$_2$CO at 120 K.

The view is parallel to the [010] crystal vector with the unit cell *a* axis horizontal, and is chosen for comparison with Figure S9. ‘A’ and ‘B’ cations (which are both high-spin) are colored white and gray respectively; anions and solvent (yellow) are de-emphasized for clarity.

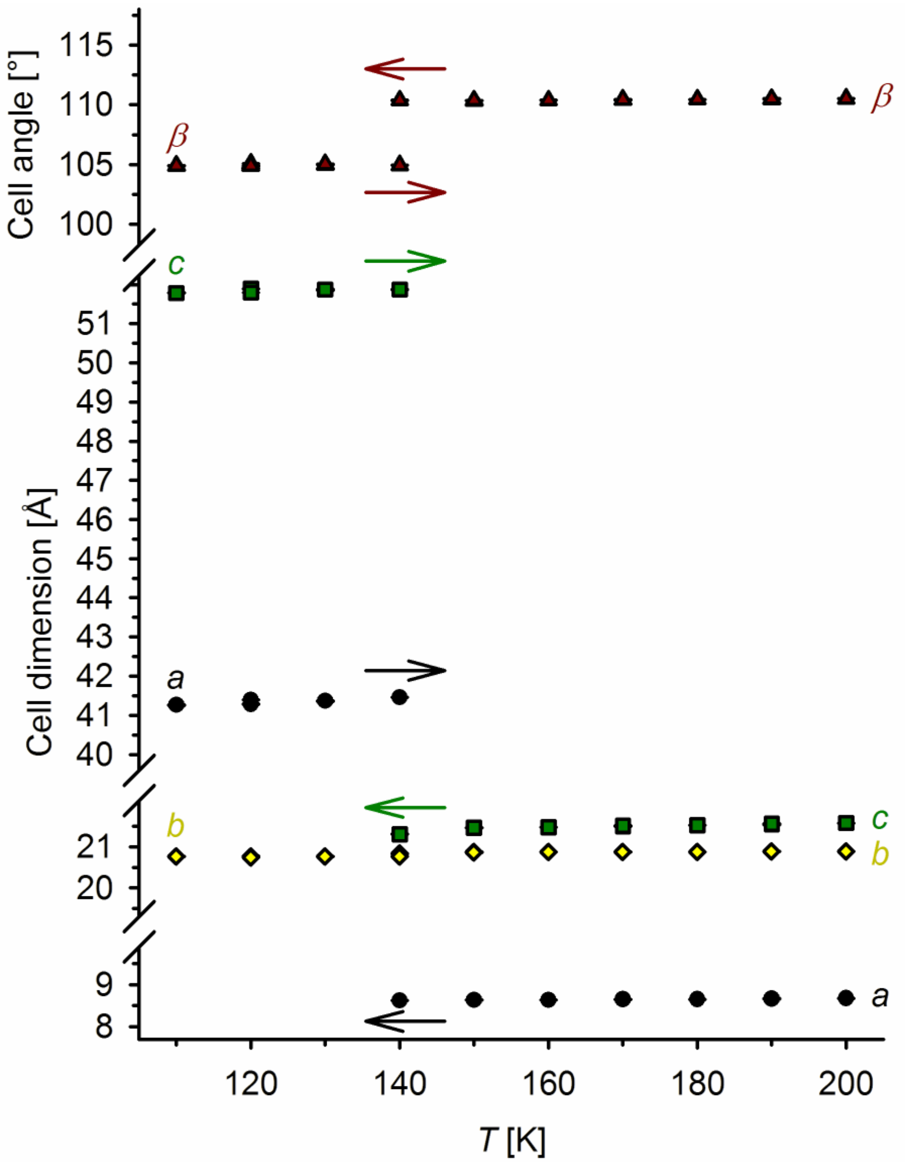

Figure S14. Variable temperature unit cell parameters for **1[BF$_4$]$_2$**·Me$_2$CO (Table S9). Data were measured on both a cooling and warming temperature ramp. Error bars are shown, but are mostly smaller than the symbols on the graph.

At 140 K, the crystal adopts phase 1 on the cooling ramp but phase 2 upon warming. That reproduces the hysteresis in the spin-transition evident in the magnetic susceptobility data (Figure 1, main article).

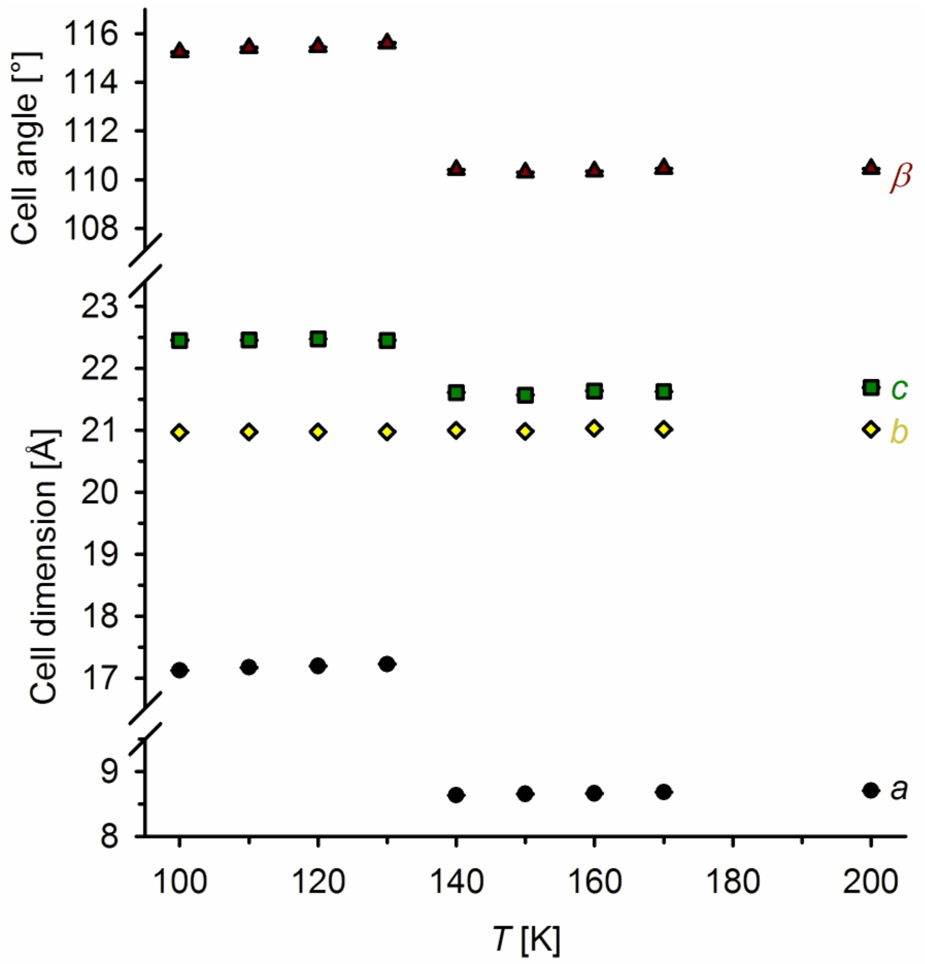

Figure S15. Variable temperature unit cell parameters for **1[ClO$_4$]$_2$**·Me$_2$CO, on a cooling temperature ramp (Table S9). Error bars are shown, but are mostly smaller than the symbols on the graph.

Table S9 Variable temperature unit cell data for **1[BF$_4$]$_2$**·Me$_2$CO and **1[ClO$_4$]$_2$**·Me$_2$CO [Å, °, Å^3] (Figures S14 and S15). Data for **1[BF$_4$]$_2$**·Me$_2$CO were measured on both cooling and warming temperature ramps.

	T / K	*a*	*b*	*c*	*β*	*V*
**1[BF$_4$]$_2$**·Me$_2$CO	200	8.6771(2)	20.895(1)	21.5792(8)	110.525(2)	3664.0
	190	8.6662(2)	20.894(2)	21.542(12)	110.479(3)	3654.1
	180	8.6522(2)	20.885(2)	21.524(12)	110.444(3)	3644.3
	170	8.6519(2)	20.880(1)	21.5035(9)	110.415(2)	3640.6
	160	8.6380(2)	20.881(1)	21.480(10)	110.383(3)	3631.7
	150	8.6296(2)	20.878(1)	21.463(10)	110.353(3)	3625.4
	140	8.625(1)	20.839(7)	21.310(5)	110.37(2)	3590.6
	130	41.368(3)	20.773(2)	51.878(3)	104.99(1)	43063
	120	41.400(4)	20.778(2)	51.900(3)	105.1(1)	43107
	110	41.264(3)	20.769(2)	51.788(3)	104.915(9)	42886
	120	41.290(3)	20.751(2)	51.796(2)	104.889(9)	42888
	130	41.372(3)	20.772(2)	51.865(3)	105.0(1)	43047
	140	41.460(4)	20.769(2)	51.874(3)	105.0(1)	43155
	150	8.6293(2)	20.865(2)	21.46(1)	110.370(3)	3622.3
	170	8.6477(2)	20.878(1)	21.51(1)	110.434(3)	3639.7
	190	8.6602(2)	20.895(1)	21.56(1)	110.506(3)	3654.3
**1[ClO$_4$]$_2$**·Me$_2$CO	200	8.708(2)	21.017(4)	21.693(5)	110.45(2)	3720(1)
	170	8.6833(2)	21.016(3)	21.6240(4)	110.458(2)	3697.2(1)
	160	8.661(2)	21.033(3)	21.636(4)	110.35(1)	3696(1)
	150	8.652(2)	20.988(3)	21.571(4)	110.30(1)	3673(1)
	140	8.634(2)	21.002(4)	21.612(5)	110.41(1)	3673(1)
	130	17.223(3)	20.978(3)	22.455(4)	115.61(2)	7316(2)
	120	17.1903(4)	20.9753(3)	22.4791(4)	115.448(2)	7318.8(2)
	110	17.168(3)	20.973(3)	22.460(4)	115.42(2)	7304(2)
	100	17.122(2)	20.967(3)	22.456(4)	115.24(2)	7292(2)

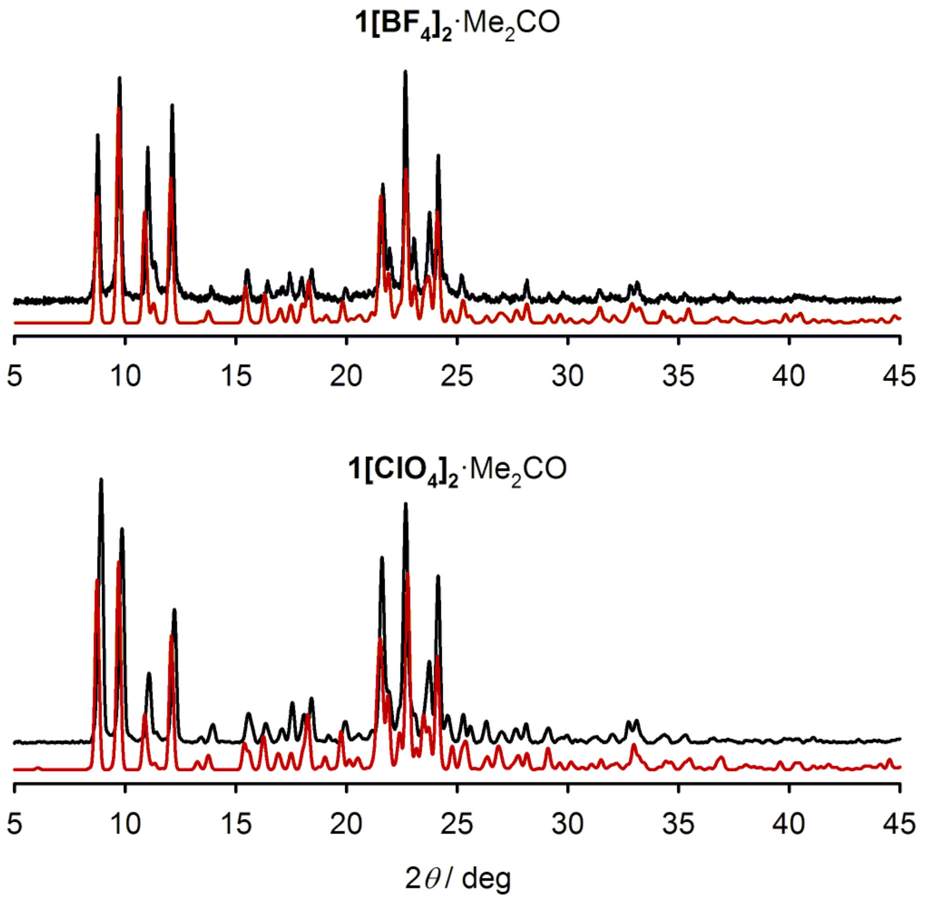

Figure S16. Experimental (black) and simulated (red) room temperature X-ray powder diffraction patterns for the complexes in this study.

References

[1] C. J. O'Connor, *Prog. Inorg. Chem.* **1982**, *29*, 203–283.

[2] C. Rajadurai, F. Schramm, S. Brink, O. Fuhr, M. Ghafari, R. Kruk, M. Ruben, *Inorg. Chem.* **2006**, *45*, 10019–10021.

[3] L. J. Kershaw Cook, R. Kulmaczewski, R. Mohammed, S. Dudley, S. A. Barrett, M. A. Little, R. J. Deeth, M. A. Halcrow, *Angew. Chem. Int. Ed.* **2016**, *55*, 4327–4331.

[4] G. M. Sheldrick, *Acta Crystallogr. Sect. A* **2008**, *64*, 112–122.

[5] L. J. Barbour, *J. Supramol. Chem.* **2001**, *1*, 189–191.

[6] O. V. Dolomanov, L. J. Bourhis, R. J. Gildea, J. A. K. Howard, H. Puschmann, *J. Appl. Cryst.* **2009**, *42*, 339–341.

[7] A. L. Spek, *Acta Cryst. Sect. D: Biol. Cryst.* **2009**, *65*, 148–155.

[8] P. Guionneau, M. Marchivie, G. Bravic, J.-F. Létard, D. Chasseau, *Top. Curr. Chem.* **2004**, *234*, 97–128.

[9] J. K. McCusker, A. L. Rheingold, D. N. Hendrickson, *Inorg. Chem.* **1996**, *35*, 2100–2112.

[10] I. Capel Berdiell, R. Kulmaczewski, M. A. Halcrow, *Inorg. Chem.* **2017**, *56*, 8817−8828.

[11] M. A. Halcrow, *Coord. Chem. Rev.* **2009**, *253*, 2493–2514.

[12] J. M. Holland, J. A. McAllister, C. A. Kilner, M. Thornton-Pett, A. J. Bridgeman, M. A. Halcrow, *J. Chem. Soc. Dalton Trans.* **2002**, 548–554.

[13] S. Vela, J. J. Novoa, J. Ribas-Arino, *Phys. Chem. Chem. Phys.* **2014**, *16*, 27012−27024.

[14] L. J. Kershaw Cook, F. L. Thorp-Greenwood, T. P. Comyn, O. Cespedes, G. Chastanet, M. A. Halcrow, *Inorg. Chem.* **2015**, *54*, 6319−6330.
